# Leaps and lulls in the developmental transcriptome of *Dictyostelium discoideum*

**DOI:** 10.1186/s12864-015-1491-7

**Published:** 2015-04-13

**Authors:** Rafael David Rosengarten, Balaji Santhanam, Danny Fuller, Mariko Katoh-Kurasawa, William F Loomis, Blaz Zupan, Gad Shaulsky

**Affiliations:** Department of Molecular and Human Genetics, Baylor College of Medicine, One Baylor Plaza, Houston, TX 77030 USA; Graduate Program in Structural and Computational Biology and Molecular Biophysics, Baylor College of Medicine, One Baylor Plaza, Houston, TX 77030 USA; Section of Cell and Developmental Biology, University of California San Diego, 9500 Gilman Drive, La Jolla, CA 92093 USA; Faculty of Computer and Information Science, University of Ljubljana, Trzaska cesta 25, Ljubljana, SI-1001 Slovenia

**Keywords:** Transcriptome, Time course, Development, Synchrony, Principal component analysis, Differential expression, *Dictyostelium discoideum*, Slime mold

## Abstract

**Background:**

Development of the soil amoeba *Dictyostelium discoideum* is triggered by starvation. When placed on a solid substrate, the starving solitary amoebae cease growth, communicate via extracellular cAMP, aggregate by tens of thousands and develop into multicellular organisms. Early phases of the developmental program are often studied in cells starved in suspension while cAMP is provided exogenously. Previous studies revealed massive shifts in the transcriptome under both developmental conditions and a close relationship between gene expression and morphogenesis, but were limited by the sampling frequency and the resolution of the methods.

**Results:**

Here, we combine the superior depth and specificity of RNA-seq-based analysis of mRNA abundance with high frequency sampling during filter development and cAMP pulsing in suspension. We found that the developmental transcriptome exhibits mostly gradual changes interspersed by a few instances of large shifts. For each time point we treated the entire transcriptome as single phenotype, and were able to characterize development as groups of similar time points separated by gaps. The grouped time points represented gradual changes in mRNA abundance, or molecular phenotype, and the gaps represented times during which many genes are differentially expressed rapidly, and thus the phenotype changes dramatically. Comparing developmental experiments revealed that gene expression in filter developed cells lagged behind those treated with exogenous cAMP in suspension. The high sampling frequency revealed many genes whose regulation is reproducibly more complex than indicated by previous studies. Gene Ontology enrichment analysis suggested that the transition to multicellularity coincided with rapid accumulation of transcripts associated with DNA processes and mitosis. Later development included the up-regulation of organic signaling molecules and co-factor biosynthesis. Our analysis also demonstrated a high level of synchrony among the developing structures throughout development.

**Conclusions:**

Our data describe *D. discoideum* development as a series of coordinated cellular and multicellular activities. Coordination occurred within fields of aggregating cells and among multicellular bodies, such as mounds or migratory slugs that experience both cell-cell contact and various soluble signaling regimes. These time courses, sampled at the highest temporal resolution to date in this system, provide a comprehensive resource for studies of developmental gene expression.

**Electronic supplementary material:**

The online version of this article (doi:10.1186/s12864-015-1491-7) contains supplementary material, which is available to authorized users.

## Background

The social amoeba *D. discoideum* exhibits a developmental program unique among model organisms [1-3]. Solitary amoebae grow vegetatively, consuming bacteria by phagocytosis. When food is exhausted, starvation triggers *D. discoideum* to cease growth and begin development. Cells signal to one another with cyclic adenosine monophosphate (cAMP) and migrate by chemotaxis into aggregation centers. Aggregates then tighten into mounds that proceed through differentiation and morphogenesis as physiologically integrated multicellular organisms.

This remarkable choreography is robust to most variations in the genetic makeup, environmental substratum, and nutritional history [4]. Some laboratory strains have been selected that grow in nutrient media, but undergo the same morphological progression as bacteria-fed amoebae when their food source is removed [5,6]. Perhaps even more impressive than watching the entrainment and chemotaxis of an entire population of cells to a centrally emitted cAMP signal, is that the multicellular organisms that arise from aggregation centers continue to develop with lock-step synchrony [1,7]. Its developmental coordination makes *D. discoideum* a desirable model for studying intercellular signaling pathways (reviewed in [8]).

Changes at the level of morphology reflect the molecular genetic physiology of the cells. The molecular milieu can be understood via complementary approaches—treatment of the entire transcriptome as a phenotype, and consideration of expression profiles of individual genes [9]. The global approach takes into account the vast amount of information available by high-throughput assays or next generation sequencing, and enables the precise grouping of molecular states even when the gross phenotype is subtle or uninterpretable. For example, Hughes and colleagues (2000) compiled the transcriptome profiles for 300 mutants and chemical treatments of *Saccharomyces cerevisiae.* Each transcriptome profile was treated as a single phenotype. This compendium of transcriptomes enabled them to discern affected genetic pathways by matching the global expression phenotypes of different mutants and treatments.

One challenge of global analyses is that these data sets contain many more variables or measurements than the number of samples or treatments to be compared. Methods to simplify the high-dimensional data so that they may be understood in more approachable two-dimensional (2D) representations include principle component analysis (PCA) and multi-dimensional scaling (MDS) [10,11]. PCA is a statistical procedure that identifies linear combinations of data variables that explain the largest proportion of variation. By charting the data according to the first two principal components we can obtain a simple 2D plot that displays the predominant relationships between samples [10]. MDS accomplishes a similar feat by arraying samples in 2D based on the similarity, or distances, between their transcriptomes [11].

Focusing the analysis at the level of individual genes is critical for assigning causative effects to specific genes or mutations. In the yeast compendium study, examining individual gene expression profiles was required to confirm various metabolic and regulatory roles [9]. Today’s bioinformatics resources, including fully sequenced, annotated genomes, and relational databases such as Gene Ontology, help illuminate the biological relevance of coincident changes in expression of many individual genes. By examining which biological process or molecular function terms are enriched for coordinately expressed genes, we can make inferences about changes in the cells that generate the observed phenotypes.

Previous *Dictyostelium* studies employed transcriptome-wide profiling to understand the global changes in gene expression that coincide with developmental progression and patterning [4,12]*.* A microarray time course study revealed that one-quarter to half of the predicted genes in the genome are developmentally regulated, with the greatest change in expression occurring upon multicellular differentiation [4]. These analyses pre-dated the completion of the genome sequencing effort. Instead of focusing on individual gene profiles, each time point was treated as a quantitative global phenotype, thereby relating the molecular genetic physiology of the cell population to the gross morphology over developmental time.

A subsequent microarray experiment examined transcriptional changes in response to cAMP signaling [12]. Rather than developing on solid support, cells were starved in shaking suspension, and cAMP was added exogenously at concentrations and with a periodicity meant to mimic natural pulsatile signaling [13-15]. Pulsing cAMP in suspension is useful for synchronizing cells for chemotaxis or aggregation assays, or for specifically studying cAMP responses. Iranfar and coworkers (2003) confirmed the involvement of several critical cAMP- regulatory and responsive genes, and identified many more by clustering co-regulated expression profiles. Later, deep RNA sequencing (RNA-seq), in conjunction with a completed genome, produced highly quantitative expression profiles for every gene model in the genome [16]. Nearly three quarters of the predicted gene models were expressed at some point in the *D. discoideum* life cycle, with many of these developmentally regulated.

We wished to build on the strengths of these previous studies, and address possible gaps in the existing data [17]. The microarray experiments were sampled every two hours, while the published RNA-seq data was sampled more sparsely, at 4-hour intervals. The cells used in the RNA-seq experiments were grown on bacteria. While this is common in the laboratory, many groups use nutrient medium as well, and thus expression data for medium grown cells would complement our understanding of the experimental system. In our study, cells were grown in nutrient medium, and either developed on solid substrate or treated with cAMP in suspension. Samples were collected for RNA-seq every hour for the first half of each time course, and every two hours thereafter. Thus we generated data with the depth and quantitative specificity of RNA-seq, at a high temporal resolution, for cells grown by a common laboratory standard.

We hypothesized that more frequent sampling would reveal unappreciated patterns in gene expression, and provide a more precise understanding of the transcriptional dynamics that govern major developmental transitions. The high temporal resolution revealed transcriptome trajectories with gradual changes interspersed with a few large shifts in global gene expression. Individual gene profiles were examined that corresponded to major changes in transcriptome and morphological progression. Further, we highlighted a selection of biological processes that turn on and off throughout development, relating population-wide physiological phenotypes with known gene functions and morphology.

## Results

### Fine-scale temporal transcriptome profiling reveals an uneven progression of developmental gene expression

We analyzed transcriptome time courses of *D. discoideum* under two experimental conditions: development on nitrocellulose filters for 24 hours (filter), and treatment with exogenous cAMP in shaking suspension for 12 hours (suspension). Samples were collected hourly for the first half of each time course and every other hour thereafter (Additional file 1: Table S1). Gene expression was analyzed by RNA-sequencing, resulting in a high dimensional data set of transcript abundance values for over 12,000 genes measured from 67 samples (Additional files 2 and 3). Over 8,000 genes in the genome were expressed and displayed some change in mRNA abundance in both experiments (Additional file 1: Figure S1, Additional files 4, 5 and 6), consistent with the results obtained by Parikh, *et al.* (2010). In that study, at 4-hour resolution, genes could be visually assigned into groups with distinct expression patterns. In the new 1-hour data, similar boundaries seemed apparent (Additional file 1: Figure S1), suggesting that developing populations of amoebae progress synchronously through discrete transcriptional stages.

**Figure 1:**
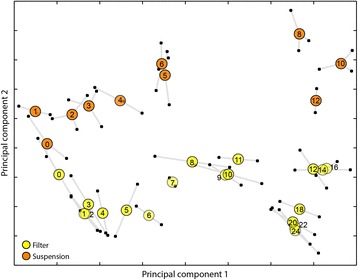
Uneven developmental progression is revealed by transcriptome time course trajectories. We developed cells on filters (yellow circles) and treated cells with cAMP in suspension (orange circles) in separate experiments. We analyzed the transcriptomes by RNA-sequencing and performed principal component analysis (PCA) to reduce the high dimensionality of the data. Both experiments were analyzed using 8,040 expressed genes intersecting the two data sets. For the filter time series, principal component 1 (PC1) and PC2 accounted for 28.6% and 19.6% of the variation, respectively. For the suspension experiment, PC1 and PC2 encompassed 31.2% and 17.3% of the variation, respectively. Plotting the second principal component (PC) (vertical axis) versus the first PC (horizontal axis) illustrates the prevailing patterns in transcriptional progression. The filter series contains two replicates of 19 time points. The suspension series contains two replicates with 10 time points and a third replicate with 9 time points (missing hour 12). For every time point we projected each sample transcriptome as a small black circle connected by whiskers to the other replicate(s). Large colored circles are placed at the center of the transcriptome projection replicates. The axes units are arbitrary.

To explore how transcriptome-wide expression changed across development, we sought to visualize the main relationships between the time-course samples. We used principal component analysis (PCA) to reduce the complexity of the data (Figure 1). The entire collection of transcript abundance data at each time point is described as one entity–the transcriptome, which represents the phenotype or physiological state of the cells at a given time. The first two principal components (PCs) accounted for nearly half of the variation in the entire data set (Filter development: PC1 = 28.6%, PC2 = 19.6%; Suspension: PC1 = 31.2%, PC2 = 17.3%). We projected the sample transcriptomes in a two-dimensional (2D) plane with axes corresponding to PC1 and PC2. The more similar the transcriptomes were at any two time points, the closer they appeared on the plots. In this simplified view, we compared transcriptome changes from one sample to the next within a time course, and compared the trajectories of transcriptional change between experiments (Figure 1). For example, the 0-hour (h) time points of the filter experiment and the suspension experiment are very close to each other, because in both cases the samples were collected from cells grown in nutrient medium before development. On the other hand, the 24-hour time point of the filter development is far removed from the 0-hour time point, consistent with a continuous accumulation of transcriptional changes during the course of development. To confirm the PCA visualization, we performed multidimensional scaling (MDS), a distinct mathematical approach that yielded a similar 2D projection in which the distance between points relates to similarity between transcriptomes (Additional file 1: Figure S2). The time course topologies were highly similar in both analyses.

Two main observations emerged from the 2D plots. First, the two treatments followed similar overall transcriptional trajectories, progressing in a mostly linear fashion (along PC1) but changing direction (along PC2) near the end of the experiments (Figure 1, Additional file 1: Figure S2). The directional spread of time points suggests that as cells proceed through development, either on filters or in suspension, their transcriptional state becomes increasingly different from the vegetative condition. The second notable feature of the 2D time course projections was uneven spacing of samples, many of which were grouped into clusters separated by gaps. In the filter series, for example, time points 9 h, 10 h and 11 h were very near one another, with 9 h and 10 h overlapping significantly. This group was separated by a considerable distance from the cluster of time points 12 h, 14 h and 16 h. We interpreted these clusters as representing stages of development in which the population-wide transcriptional state changed rather slowly. The gaps between clusters signified concerted changes in gene expression between developmental stages (Additional file 1: Supplementary results, Additional file 1: Figures S3–S5). Thus the overarching structure of the transcriptome time courses could be summarized as groups of time points with gradually changing cellular physiologies interspersed with dramatic shifts in gene expression. This description is consistent with the clustering of transcriptome samples based on Spearman’s correlations (Additional file 1: Figure S6, S7). Together, these findings suggest that the rate of development as a whole is uneven–some stages progress faster than others.

### Transcription profile inference depends on temporal resolution

**Figure 2:**
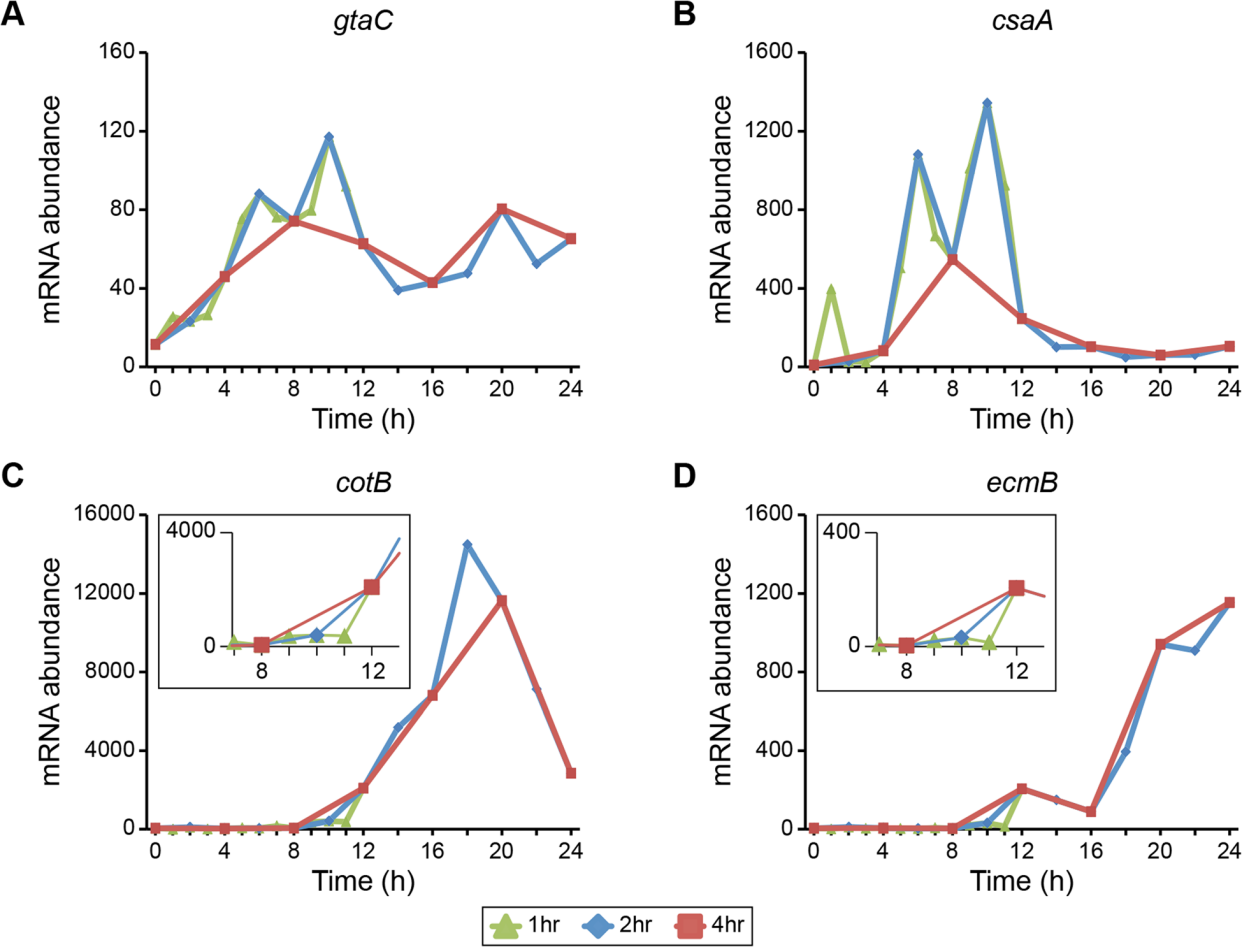
Temporal resolution affects the interpretation of transcription profiles. The standardized mRNA abundance of four developmentally regulated genes (y-axis) is plotted versus time (hours, x-axis). Data are from the filter development experiment. For each gene—*gtaC* **(A)**, *csaA* **(B)**, *cotB* **(C)**, and *ecmB* **(D)**—expression values are included for time points at 1-, 2- and 4-hour intervals, as indicated in the legend below the figure. Each data point represents the average of 2 independent biological replicates. The y-axis scale varies between plots. The insets in **(C)** and **(D)** highlight the 8 h – 12 h time frame.

Previous RNA-seq studies described mRNA abundance with rather sparse sampling [16,18], so it was interesting to examine the behavior of genes with known functions at higher temporal resolution. We selected 32 well-characterized, developmentally regulated genes (Additional file 1: Table S3) and plotted their mRNA abundance versus time. First we asked whether the inferred transcription profiles were sensitive to the frequency of sampling, i.e., 1-, 2- and 4-hour time intervals (Figure 2, Additional file 1: Figures S8, S9 and S10). Generally speaking, profiles that changed monotonically or with a simple “on then off” modality were fairly robust to sampling interval, while others displayed more complex patterns only discernible at higher temporal resolution.

The master transcriptional regulator *gtaC,* and several of its putative target genes involved in aggregation (such as the cell-cell adhesion gene *csaA*), looked markedly different at different time scales (Figures 2A, B). The shape of the expression curve, as well as amplitude and timing of peak expression, varied between 1-hour and 4-hour sampling. However, 2-hour sampling was nearly identical to the 1-hour curve, suggesting that 2-hour intervals are sufficiently frequent to accurately describe population-level changes in mRNA abundance.

Identifying the timing of up- or down-regulation can impact the interpretation of gene function or cellular dynamics. The genes *cotB* and *ecmB* are often used to mark the differentiation of prespore and prestalk cell-types, respectively [19]. At 4-hour resolution, the up-regulation of these genes could be inferred to begin sometime between 8 h and 12 h, whereas 1-hour sampling placed their up-regulation precisely between 11 h and 12 h (Figures 2C, D). These analyses revealed the expression of cell-type specific genes was coincident with the major transcriptome shift at that time.

For other genes, such as the chemotaxis-related *phdA*, the 4-hour sample frequency captured the overall shape of the profile, even if small changes were missed (Additional file 1: Figure S8). Likewise, 4-hour sampling of the allorecognition determinants *tgrB1* and *tgrC1* was consistent with the overall shape, but not the precise timing of expression changes, at 1-hour frequency (Additional file 1: Figure S8). Still others, *sigB* for example, were equally well described at all time scales we tested (Additional file 1: Figure S9).

### Transcription profiles during early development reflect differences in exposure to cAMP and physical contact

**Figure 3:**
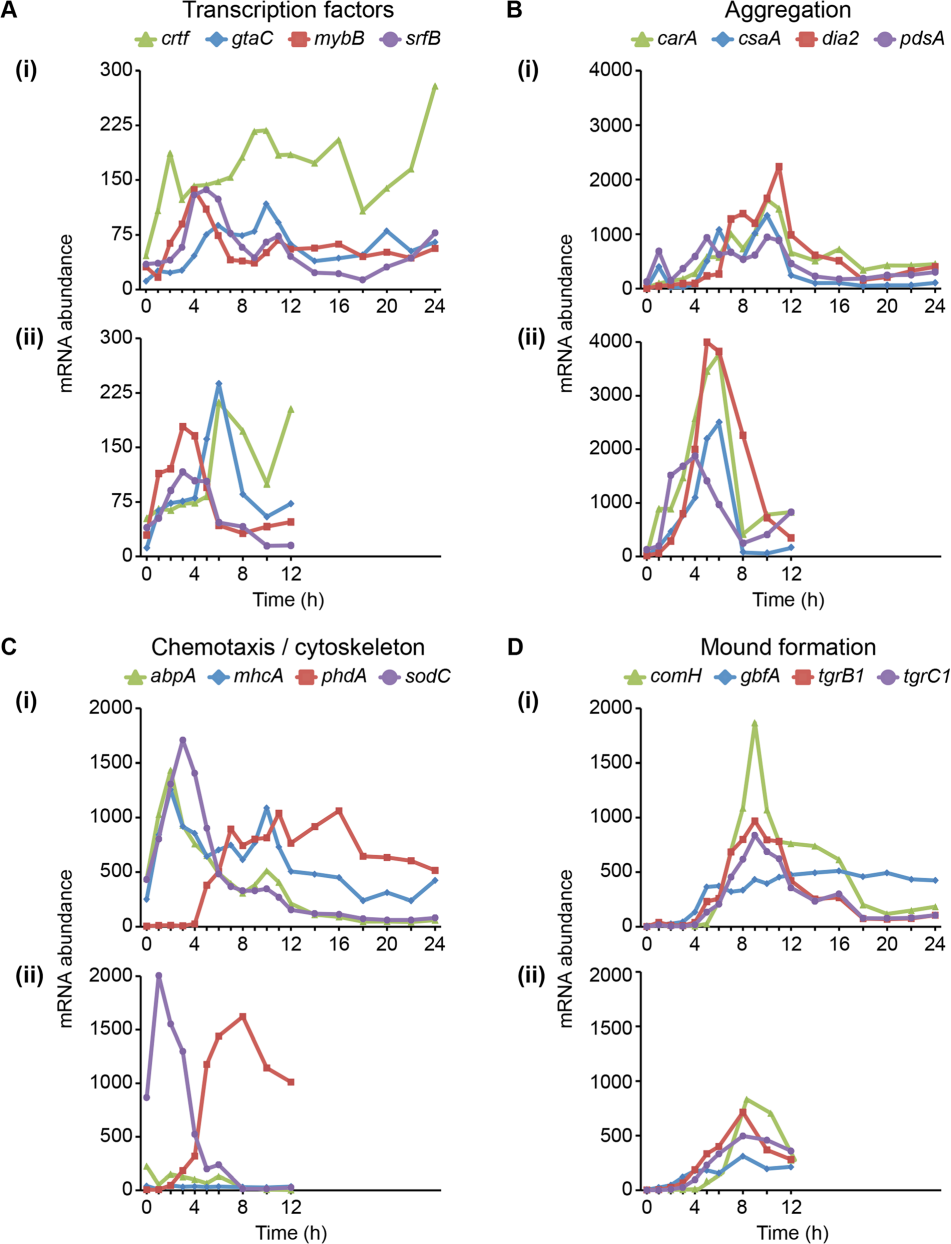
Early development mRNA abundance reflects cAMP experience and physical contact. The standardized mRNA abundance of genes involved in early development (y-axis) is plotted versus time (hours, x-axis). We grouped the genes based on general functional class or developmental process in which they are involved, as indicated above the panels. Legends above the panels indicate the gene names. For each gene category **(A – D)**, data from the filter experiment are shown in the top panel (i) and data from the suspension experiment in the bottom panel (ii). Within each category, the vertical axis scale is constant to facilitate direct comparisons between filter development and liquid suspension. Between categories, the vertical axis scale varies to accommodate different levels of gene expression. Each data point represents the average of 2–3 independent biological replicates as indicated in Figure 1.

We compared the transcription profiles of selected genes expressed at 0 h–12 h between cAMP treatment in suspension and development on filters (Figure 3, Additional file 1: Table S3). Many of these genes displayed qualitatively similar profiles between treatments, though interesting differences were observed as well. Several transcription factors (TFs) (reviewed in [20]) were initially up-regulated during starvation and the beginning of development (Figure 3A). The precise timing of onset and peak expression varied between the experimental treatments, however. The genes *mybB* and *srfB* peaked at 3 h in the suspension series, and at 4 h or 5 h on filters; *crtf* and *gtaC* peaked at 6 h in the suspension, and at 10 h in the filter data. This temporal delay in peak expression on filters was consistent with other groups of surveyed genes.

Cells in suspension were treated with 30 nM exogenous cAMP, applied at a fixed 6-minute period to all cells in the flask equally. Thus developmental processes that require pulsatile cAMP or entrainment to this signal might be expected to respond more rapidly. Indeed, the most pronounced difference in transcript abundance between treatments was observed among genes that mediate aggregation towards pulsatile cAMP [12] (Figure 3B). Specifically, *carA*, *csaA, dia2 and pdsA* were expressed earlier, more rapidly and to higher levels in the first 6 hours of the suspension treatment than during any time in the filter development. The lower amplitude on filters might be attributable to spatial variation in a field of cells, which likely encounter local differences in cell density and substrate topography, thus resulting in less uniform cAMP signaling and slower entrainment towards synchronicity.

The delivery of cAMP to suspension cells was designed to mimic conditions in actual development—starvation, followed by cAMP pulsing, then by high constant levels of cAMP. We asked whether the effects of changing the cAMP regime from pulsing to constant levels were indeed mirrored in the filter-developed samples. In the suspension samples, expression of aggregation genes peaked at 6 h, followed by a sharp down-regulation between 6 h and 8 h (Figure 3B [ii]). The downturn corresponded to the change from pulsatile to bulk cAMP. A similar down-regulation in transcript abundance was observed in the filter samples between 10 h and 12 h (Figure 3B [i]). During this time interval, cells on filters developed into tight aggregations and mounds, where they were expected to experience higher levels of accumulated cAMP. Together these data further indicate that the exogenous cAMP regimen elicits a transcriptional response highly similar to early development on solid support. This result is consistent with the earliest comparisons between cells signaling in suspension and those undergoing development [21].

Developmental gene expression not only depended on cAMP, but also on physical cues such as contact with the substratum. Pulsed cells displayed differential expression of the chemotaxis-related genes *sodC* and *phdA,* but not of the cytoskeletal genes *abpA* or *mhcA*, which are also required for motility [22-26] (Figure 3C [ii]). All four of these genes were differentially regulated in the filter-developed cells (Figure 3C [i]). The failure to turn on these cytoskeletal genes in suspension represents a major difference in transcriptional response between the two experimental treatments.

In order for *D. discoideum* to develop beyond the aggregation stage into mounds, amoebae must clear a checkpoint established by the allorecognition genes *tgrB1* and *tgrC1* [27,28]. The *tgr* genes were expressed nearly identically between treatments, though they accumulated to higher levels on filters (Figure 3D). Two transcription factors involved in the transition from aggregates to mounds, *gbfA* and *comH* [29,30]*,* also displayed temporally consistent expression between treatments, though *comH* abundance spiked higher on filters. Thus the expression of genes required for mound formation and tissue-grade organization was largely robust to differences in cAMP regimen and substratum contact.

### Late development mRNA abundance marks timing of cell differentiation and morphogenesis

**Figure 4:**
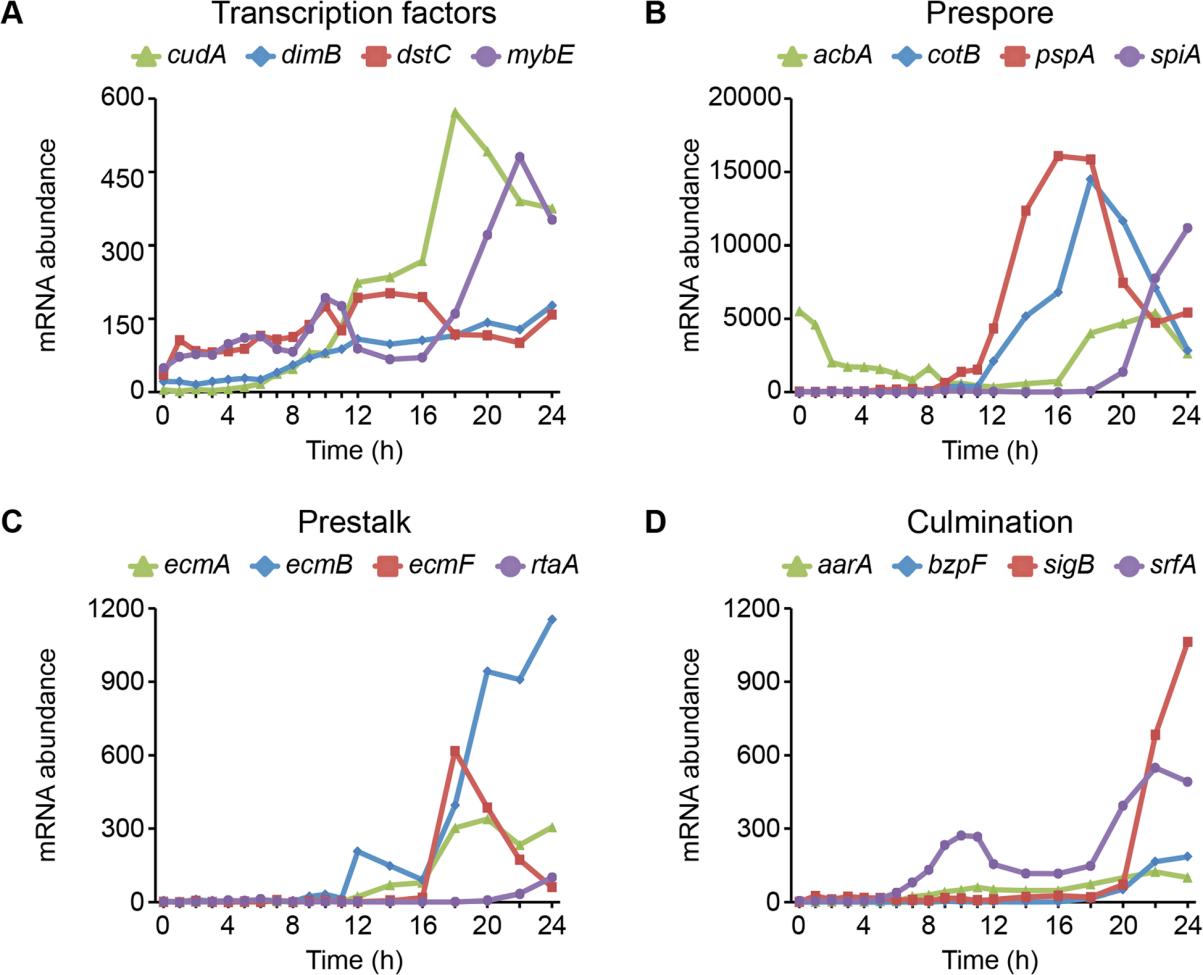
Cell-type specification coincides with major changes in transcriptome phenotype. The standardized mRNA abundance of genes involved in later development (y-axis) is plotted versus time (hours, x-axis). Data are from the filter development experiment. We grouped the genes based on general functional class or developmental process in which they are involved **(A – D)**, as indicated above the panels. Legends above the panels indicate the gene names. The vertical axis scale varies to accommodate different levels of gene expression. Each data point represents the average of 2 independent biological replicates.

We examined the transcription profiles of select genes involved in later cell-type differentiation, morphogenesis, and culmination (Figure 4), searching for shifts in mRNA abundance coincident with major developmental transitions. We first considered the expression of several transcription factors that regulate late developmental events [20]. All of the plotted TFs began to accumulate transcripts gradually prior to the multicellular transition. At 16 h, *cudA* and *mybE* were up-regulated sharply, while *dimB* and *dstC* continued on a more graded trajectory (Figure 4A). The difference in temporal dynamics of TF expression might reflect the nature of regulatory interactions with their targets. Even at peak expression, the mRNA abundance of the transcription factors was lower than that of other classes of genes, especially genes whose products contribute to structural composition. This finding is consistent with previous observations about the relationships between level of transcript abundance and predicted gene function [16].

Examples of highly expressed structural genes included the prespore genes *cotB* and *pspA* [31]. Both showed rapid accumulation beginning between 10 h and 12 h, leading to high levels between 16 h and 18 h (Figure 4B). Highlighting its utility as a sporulation marker [32,33], *spiA* was up-regulated between 18 h and 20 h, and accumulated rapidly through 24 h. The expression profile of *acbA* was consistent with its known housekeeping function during vegetative growth, and a signaling function in terminal spore differentiation [34,35] (Figure 4B). Expression of the prestalk genes *ecmA, ecmB and ecmF* ([19,36], could be detected as early as 12 h, but were up-regulated more dramatically between 16 h and 18 h (Figure 4C). Structural prestalk transcripts accumulated to levels an order of magnitude less than those of the surveyed prespore genes, probably due in part to the 1:4 ratio of prestalk to prespore cells in the multicellular body. The gene expressed the latest among this set was *rtaA*, thought to indicate the differentiation of the prestalk subset pstU cells [37]. In general, the initial up-regulation of cell-type specific genes between 10 h and 12 h, and 16 h and 18 h, coincided with the gaps and rapid differential expression intervals in Figure 1 and Additional file 1: Figure S5.

Lastly, various transcription factors and membrane-associated proteins involved in culmination and fruiting body formation were expressed in the last 4 to 6 hours of development (Figure 4D). These data suggest that our samples followed stereotypic developmental progression. Prespore and prestalk cell types could be differentiated by changes in expression between hours 11 and 12, coincident with the transition between tight aggregate and mound stages of development. Specific subtypes of cells continued to differentiate with the expected timing through the slug and culmination stages.

### Overlapping biological processes are regulated at variable time scales

To explore what biological processes are regulated during development, we tabulated lists of differentially expressed genes counted in each k-hop comparison throughout development (Additional file 1: Figure S5, Additional file 7) and performed Gene Ontology (GO) enrichment analysis to identify biological processes up- or down-regulated at various intervals in development (Additional file 8). Rather than treating GO enrichment as a definitive result, we approached this analysis as an exercise in hypothesis generation.

**Figure 5:**
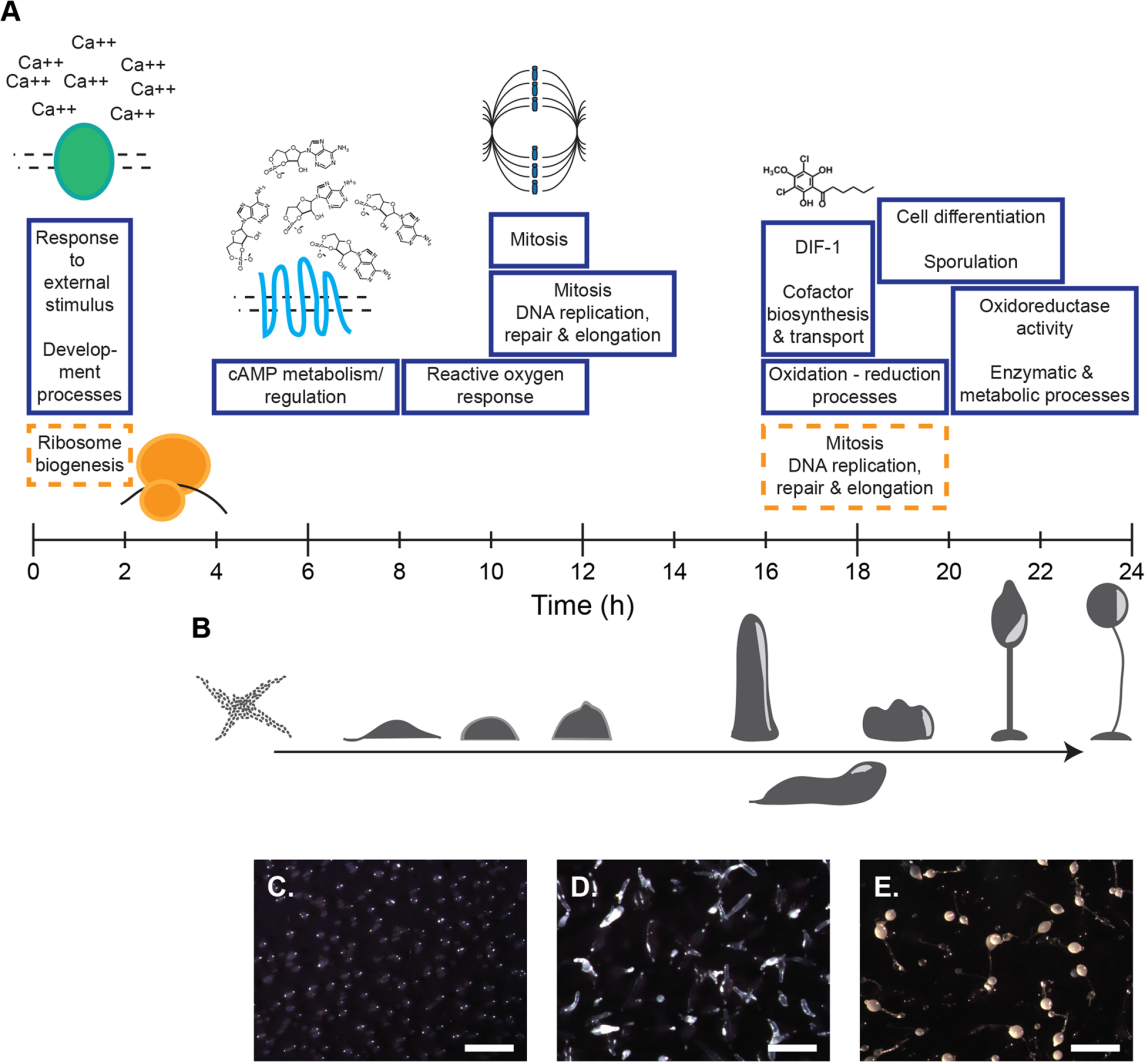
Differential expression of functional modules during development. For the filter development experiment, we analyzed the GO term enrichment for all DE genes in the rapid and gradual k-hop intervals at each time point (see Additional file 1: Figure S5A). **(A)** Selected terms are illustrated over the 24-hour developmental timeline. Solid blue boxes correspond to up-regulated genes and dashed orange boxes to down-regulated genes. The width of each box indicates the time delta in which the corresponding GO term is enriched. **(B)** A cartoon of developmental morphogenesis, with micrographs corresponding to **(C)** 12-hour mounds, **(D)** 16-hour slugs, and **(E)** 24-hour fruiting bodies. Micrograph scale bars represent 1 mm.

By comparing some of these processes with the developmental timeline, we were able to postulate which cellular and biochemical activities align with morphological progression (Figure 5). Of the subset of enriched GO terms illustrated here, some were well known and expected. We observed an expected reduction in ribosome biogenesis at 0–2 h, an increase in cAMP metabolism mRNA abundance at 4–8 h, increased Differentiation Inducing Factor 1 (DIF-1) biogenesis between 16–18 h and an increase in sporulation morphogenesis mRNA abundance toward the end of development. We also observed others, such as reactive oxygen response at 8-12 h, that have received less attention in the field.

The rapid intervals of highest synchrony were 10 h–12 h and 16 h–18 h. These contained DE genes enriched for GO terms related to mitosis, and to organic signaling molecule pathways and co-factor synthesis and transport, respectively (Figure 5A). GO terms related to managing cellular energetics and redox chemistry, such as reactive oxygen species and oxidation-reduction processes, were up-regulated at more than one interval over the course of development. The two intervals of major down-regulation included enriched terms pertaining to ribosome biogenesis from 0 h–2 h, and genes associated with mitosis and DNA replication, repair and elongation between 16 h and 20 h. Many genes in this latter set were among those up-regulated between 10 h and 12 h, during which time the prespore cells, already in G2-phase, undergo cell division and transition to G1-phase [38-40].

Visual inspection of development on filters confirmed the highly reproducible morphological progression. We observed tight aggregates at 10 h–14 h (Figure 5C), fingers at 16 h–18 h (Figure 5D) and fruiting bodies at 22 h–24 h (Figure 5E). The uniformity of the filters underscored the synchrony of structural changes within and between multicellular bodies in a developing field.

## Discussion

### Cellular transitions are reflected in unevenly spaced transcriptome trajectories

*Dictyostelium discoideum* development requires coordination between many tens of thousands of individual cells coalescing to form multicellular organisms [1,3]. The developing field continues to display remarkable synchrony within and between distinct multicellular structures as they pass through each morphological stage. Recent RNA-seq time course studies offered significant gains in detail over previous microarray efforts, but lacked the temporal resolution to address how changes in gene expression precisely correspond to developmental progression [4,12,16-18].

The data generated in our current study complement previous efforts, clarifying the relationships between transcriptome and morphology during development, and greatly expanding the community’s resources to study developmental gene expression. We benefited from the quantitative depth of RNA-seq and sampled every hour or two, the highest temporal frequency of any study in the field to date. But our analysis also surpasses previous work in our ability to link detailed mRNA abundance profiles to global trends, biological process enrichment and morphological progression. We found that the developmental transcriptome is characterized by intervals of gradual change punctuated by concerted shifts in gene expression. Consistent with previous studies, the major changes in transcriptome state corresponded to starvation, multicellular differentiation and culmination.

The transcriptome is only one aspect of development, however. Global patterns of gene expression alone do not reveal the regulation or governance of the stereotypic developmental progression. Rather, it may be more useful to think of the transcriptome as a quantitative phenotype, a read-out of the population average of cellular behavior or physiology [4,41]. Intervals of gradual transcriptional change might result from the need to entrain a dispersed population to a soluble signal [7,8,15]. For example, the differential expression of genes during cAMP-mediated aggregation was relatively gradual, possibly reflecting heterogeneity in the sensing, processing and propagation of this signal in the developing field of cells. This interpretation is supported by the observation of nearly twice as many DE genes up-regulated in the suspension treatment relative to the filter development experiment. In suspension the application of cAMP was uniform, and the DE response more immediate and intense than on the spatially heterogeneous filters.

Times of rapid mRNA accumulation, alternatively, might fulfill a need to quickly generate sufficient macromolecules, i.e. signaling compounds such as DIF-1 or structural components like spore coat proteins, before cells transition to a new identity [42-44]. The two most predominant instances of differential up-regulation in our data coincided with the transition from aggregates to mounds and the transition from finger to culminate stage. Both of these morphological transitions involve cell-type specification, and thus the adoption of specific molecular identities by large subsets of cells in the population (reviewed in [19]). Another possibility is that rapid shifts in mRNA abundance occur once a critical proportion of the cell population reaches a transcriptional checkpoint. If this were the case, one would expect the population average transcriptome to change at some background pace until the checkpoint threshold is reached, then the cells quickly transition to a new state.

### Temporal coincidence of mRNA abundance associated with multicellularity, energetics and signaling

We expected to see considerable transcriptional changes at the beginning of the time series. Much of this should reflect the starvation response, while some undoubtedly results from the experimental manipulation of the cells. Significant down-regulation of ribosome biogenesis genes was consistent with known physiological changes characteristic of starvation [45,46], and suggests that other processes enriched for DE genes might be part of the starvation response as well. Enrichment of up-regulated genes confirmed that cells are primed from the onset of starvation to receive external cues, including calcium and cAMP [15,47,48]. These results corroborate published findings that some of the core cAMP relay genes, such as *acaA* and *carA*, are initially expressed prior to cAMP pulsing, and then accumulate further in response to continued cAMP exposure [12,49].

The timing of the most dramatic up-regulation of mRNA abundance took place between 10–11 h and 12–14 h of development. This interval appears as the largest gap on the 2D plots. Comparing these data with observed morphological changes indicates that the largest change in transcriptome state of any time in development coincides with the transition from aggregates to multicellularity. Van Driessche and colleagues (2002) demonstrated that this major transcriptional shift at the transition to multicellularity is robust to both amoebae strain and nutritional history. This timing also overlaps with a major burst of mitochondrial DNA replication that occurs prior to prespore cell division [38-40,50]. Other reports argue that nuclear DNA replicates during this time as well, although newly synthesized DNA labeled with 5-bromo-2-deoxyuridine has been observed only in the mitochondrial fraction [50,51]. Differentially expressed genes between these time points are strongly enriched for GO terms related to DNA replication, mitosis and associated processes and reactive oxygen response.

When *D. discoideum* begin to develop, they no longer consume food. Thus the energy required for aggregation and multicellular development must come from internal stores [52]. Cellular energetics requires considerable mitochondrial and cell membrane activity [53-57]. In *Dictyostelium,* mitochondria genome encoded transcripts are not poly-adenylated, and therefore cannot be quantitatively analyzed in our data due to the poly-A selection used for mRNA purification. However, prior work suggests that some of these genes are essential for development and differentiation [57]. In addition to potentially causing cellular harm, byproducts of respiration such as reactive oxygen species (ROS), may play an active role in developmental signaling [58]. One recent study in mice concluded that mitochondria-derived ROS were critical mediators of epithelial and hair follicle differentiation [59,60]. During early development in *D. discoideum*, ROS are thought to play a signaling role related to cAMP-mediated aggregation [61]. Ectopic superoxide scavengers or over-expression of a dismutase enzyme inhibited aggregation. This result is consistent with the observed differential expression of the superoxide dismutase gene *sodC* during aggregation.

We found that genes related to ROS and redox chemistry were differentially expressed at multiple time points, namely around the time of mtDNA replication and mitosis and later during the generation of soluble signaling macromolecules such as DIF-1 (16 h — 20 h). Among the genes turned on was the prespore specific catalase *catB*, a target of SrfA that catalyzes the decomposition of hydrogen peroxide and is essential to late development and spore viability [62,63]. Of the seven superoxide dismutase genes annotated in the *D. discoideum* genome, *sodD* was differentially expressed during this later stage of development as well. The overlap of redox processes with the expression of various cell-signaling pathways in *D. discoideum* could indicate that ROS modify signaling macromolecules in some functional way [58]. Interactions between ROS and transcription factors are also well documented in various higher eukaryote models [64]. These possibilities remain to be tested in *Dictyostelium*, and might represent interesting examples of evolutionary conservation in the interplay between metabolism and developmental signaling.

### Synchrony between developing structures

Development in *D. discoideum* is so well synchronized from aggregation through fruiting body maturation that Sydney Brenner, tongue-in-cheek, dubbed it “molecular fascism” [65]. The entrainment of aggregating cells to pulsatile cAMP is a well-characterized process [8,15]. Numerous pathways also have been identified that govern multicellular signaling (reviewed in [8]). Some of these are mediated by membrane bound proteins and require cell-cell contact [27,28]. Others use soluble molecules such as various polyketides that direct prestalk and prespore fates, and spore maturation, i.e. DIF-1, Spore Differentiation Factor 1 (SDF-1) and SDF-2 [66,67]. Additional signals are relayed by gamma-aminobutyric acid (GABA), glutamate, and steroids [68-70].

Despite a fairly robust picture of signaling within developing structures, little is known about how distinct multicellular structures arising from the same population develop in tight lock-step with one another. Perhaps the initial cAMP-mediated coordination sets a precise molecular clock that keeps time in each aggregate through late development. Alternatively, multicellular structures might be kept in sync by shared environmental cues such as ammonia, alkalinity, moisture and light [71-74]. Another possibility still is that the organisms signal to one another using volatile compounds. For instance, ROS have been implicated in regulating long distance signaling between developing leaves in *Arabidopsis* [75]. These molecules conceivably could be involved in synchronizing culmination and fruiting body formation between multicellular structures. Alternatively, the *Dictyostelium* genome encodes numerous enzymes that synthesize aromatic compounds, several of which are developmentally regulated [76,77]. Perhaps some of these produce cues that help synchronize late development. The transcriptome time course data enable us to determine which candidate genes are expressed at the appropriate times to contribute to developmental coordination. Understanding the molecular genetic bases for late developmental synchrony, and the evolutionary fitness benefits of such a system, offer promising opportunities for future research.

### Comparisons between experimental treatments

Directly comparing the transcriptome dynamics between cells developed on filters with those treated with cAMP in suspension yielded some interesting biological observations and useful technical information. The inclusion of the suspension data provided a time series with defined inputs that mimicked morphological or behavioral transitions. These proved consistent with our inference that clustering of transcriptome trajectory points represented gradual change in mRNA abundance at the population level, while gaps in the trajectory reflected major physiological changes. Despite the major differences in assay set-up, the suspension transcriptomes represented the progression of early development quite well [21].

Many genes show the same profiles in cells pulsed in suspension as cells developed on filters. The cumulative data increases our confidence in the patterns, and tell us that there are few significant signals that the cells pulsed in suspension do not get in the first half of development. Further, the high temporal resolution of these data allows us to recognize more clearly those genes that start to accumulate mRNAs at the same time. Going forward, researchers who rely on suspension cAMP treatment to prime cells for chemotaxis or other developmental assays may benefit from knowing exactly how genes and processes behave relative to solid-support development.

The major difference in transcriptional response between experiments was the differential expression of approximately twice as many genes in the suspension treatment than the filter treatment. As mentioned above, we suggest that this result reflects the uniformity of cAMP experience among the population of cells in suspension, both in terms of the concentration and the periodicity of the signal. Further, the transcriptional patterns were more advanced in cAMP pulsed cells relative to filter-developed cells by about 4 hours. Could this speed up be the consequence of having full amplitude pulses starting at 2 h in suspension but maybe not until 6 h on solid support? On filters the cells have to entrain each other for the whole filter to get a full amplitude pulse. Or could it be the result of having regular 6-minute pulses when cAMP is added exogenously, while on filters the signal might be more irregular? The regularity and amplitude of the cAMP pulse could affect the level of nuclear active GtaC, which would have major consequences for the transactivation and repression of certain genes [78]. Consistent with this hypothesis, we observed roughly 2-fold more *gtaC* mRNA in the suspension treatment. The four “aggregation” genes shown in the expression profile panels are all putative transcriptional targets of GtaC [78,79], and also displayed 2-fold higher transcript abundance in suspension.

Further analyses of these data may focus on identifying additional co-regulated transcriptional regulators and target genes. The enhanced temporal resolution of these data provides more informative transcription profiles than previous studies. Perhaps clustering of genes by expression pattern will yield improved hypotheses regarding shared regulation and function. As future studies examine the phenotypic consequences of transcription factor knockout mutations, as well as the binding specificity of important transcriptional regulators, these data will serve as a critical reference point for inferring regulatory interactions. To facilitate exploration by the larger community, user-friendly analysis tools for these data are available at dictyExpress [www.dictyExpress.org], and the complete time series data sets are accessioned at the NCBI Gene Expression Omnibus [GEO: GSE61914; http://www.ncbi.nlm.nih.gov/geo/query/acc.cgi?token=glcluooejnuhdmd&acc=GSE61914].

## Conclusions

Our data provide detailed molecular support for the established model of *D. discoideum* development as a series of coordinated cellular and multicellular activities. The population-average transcriptome changed rather gradually within the field of cells during cAMP relay, and at times within multicellular bodies, such as mounds or migratory slugs, that experience both cell-cell contact and various soluble signaling regimes [8]. Concerted transcriptome shifts marked the transition to multicellularity and culmination. From these observations we conclude that developmental gene expression in *D. discoideum* progresses unevenly. We further propose that the data reported here provides a substantial resource for future studies in developmental gene regulation, signaling and mechanisms of intercellular synchrony.

## Methods

### Growth, development, cAMP treatment and cDNA library preparation

For both filter and suspension development experiments we used *Dictyostelium discoideum* strain AX4 grown in HL-5 nutrient medium in shaking culture at 22°C to mid-log phase. We developed cells on nitrocellulose filters (5 × 10^7^ cells per 5 cm filter) saturated in PDF buffer by standard methods, detailed in [18]. Every hour from zero to 12, and every two hours thereafter, we scraped the cells from a single filter into 1 mL Trizol reagent (Life Technologies), vortexing to disrupt the cells and homogenize the solution. Samples were immediately stored at -80°C. In total, we collected 19 time points from two independent biological replicates on different days, yielding 38 samples. We developed cells in suspension with exogenous cAMP as in [12]. Briefly, cells were washed, resuspended and shaken at 125 RPM for a total of 12 hours (h). We began pulsing 30 nM cAMP at the 2 h time point with a six minute period until 6 h. From 6 h on, we applied 300 μM cAMP every two hours. We harvested 5 × 10^7^ cells every hour from 0 h to 6 h, and every two hours thereafter until 12 h. Cells were pelleted, resuspended in 1 mL Trizol reagent, disrupted by vortexing, and immediately stored at -80°C. In total, we collected 10 time points from three independent biological replicates on different days, except for hour 12 in replicate 3, yielding 29 samples. We extracted, precipitated and resuspended total RNA in 10 mM Tris–HCl (pH 7.5). Next we isolated and fragmented mRNA, and constructed multiplexed cDNA libraries according to [80].

### Sequencing, mapping and read count standardization

We sequenced the cDNA libraries by high throughput Illumina Genome Analyzer II with read length of 50 base pairs (bp). We mapped the resulting FASTQ files using bowtie, version 0.12.7 [81], allowing for single hits (−m 1). Unmapped reads were trimmed by 2 bp and remapped iteratively up to five times. The total number of uniquely mapped reads constituted a gene’s transcript abundance. We standardized the abundance levels as reads per kilobase per million (RPKM) by accounting for the mapable lengths of genes and the total number of mapped reads in a given RNA-seq run. The data have been accessioned at the NCBI Gene Expression Omnibus [GEO: GSE61914]. The sampling effort and sequencing output are summarized in Additional file 1: Table S1.

### Transcriptome trajectory analysis by PCA, MDS and linear regression

We filtered the experimental transcriptome data for genes that had fewer than 30 reads at all time points, leaving 8,766 genes in the filter development set and 8,593 in the cAMP suspension set, with 8,040 genes intersecting both. We implemented principal component analysis (PCA) and multidimensional scaling (MDS) algorithms using the Orange data-mining suite with default parameters [82]. PCA enumerates vectors that capture the variation among the datasets, while MDS returns a measure of distance, or similarity, between datasets (i.e. time points). For PCA, we transformed an input data matrix of size 69 × 8040 (samples × genes) into a matrix of size 69 × 2, where each of 69 sample cell populations was characterized by the first two principal components. For MDS, transcriptional distance was estimated using (1 minus the absolute value of a Spearman’s rank correlation). The MDS projection positioned each sample transcriptome in a two-dimensional plot such that the Euclidean distance between any two points is proportional to the distance between two associated transcriptional profiles. We then developed models to predict the developmental time of a cell population given its transcriptional profile. Linear regression was implemented via scikit-learn [83]. Models were constructed (or trained) using data from one experiment (e.g., filter) and the prediction accuracy was tested on the data from the other experiment (e.g., suspension). Accuracy was assessed by measuring the Spearman rank correlation between predicted and true development times of the cell populations in the test set, and, due to differences in the time scales, taking into account the developmental order of samples rather than the actual prediction of developmental time.

### Differential expression analysis

We analyzed differential expression (DE) using baySeq (R package version 1.16.0) [84], comparing the expression of each gene at each time point versus all other time points. We selected a false discovery rate (FDR) threshold ≤ 0.01 to identify genes differentially expressed in each time point comparison. We used a k-hop approach to count the number of DE genes in all k = 1-, 2-, 3- and 4-hour windows. The direction of fold change was also determined for each gene at each time point comparison. For up-regulation, we counted DE genes looking back from each reference time point, i.e. 4-hour versus 3 h, 2 h, 1 h and 0 h. We counted down-regulated genes looking forward in time, i.e. 0-hour versus 1 h, 2 h, 3 h and 4 h. Genes were only counted once per reference time point for the smallest time delta in which it appeared. For example, if a gene was DE between 4 h and 3 h, as well as between 4 h and 2 h, we counted it in the 1-hour bin.

### Correlation analyses, cluster visualizations and gene ontology enrichment

We calculated Spearman rank correlations and performed hierarchal clustering for heatmaps and dendrograms using the visual programming environment Orange [82]. Approximately Unbiased (AU) p-value scores were calculated for the dendrograms by multiscale bootstrap resampling using the pvclust package in R [85]. We tabulated a list of all DE genes in each 1- to 4-hour window and analyzed the gene ontology term enrichment using both the GO widget in Orange as well as the topGO R package version 2.14.0, with a p-value threshold of 0.05. Significantly enriched terms were selected based in part on the number of genes contributing to enrichment, and in part on the annotations.

### Data availability

The additional material includes the Additional file 1: Figures (S1 to S10) and Tables (S1 and S2) referred to in the main text. We have also included additional data files as downloadable spreadsheets. These contain the read counts, gene lists and other data analyses described in text. Please see the section “Description of Additional Data Files” below for more information. The read count data from Additional files 2 and 3, as well as individual sequencing reads files, have been accessioned at the NCBI Gene Expression Omnibus [GEO: GSE61914; http://www.ncbi.nlm.nih.gov/geo/query/acc.cgi?token=glcluooejnuhdmd&acc=GSE61914]. These data may also be viewed and analyzed using the dictyExpress toolkit [www.dictyExpress.org].

## Additional files

Additional file 1:
**Contains the supplementary results, Additional file** **1** **: Figures S1 – S10 and Additional file** 1 **: Tables S1 – S3 as referenced in the text.**
Additional file 2:
**Contains three worksheets with data from the filter development experiment: Worksheet 1—“filter_raw”—contains the raw read counts for all gene models (column header = “locus_tag”) in the** ***D. discoideum*** **genome.** Column headings indicate the time point, followed by the suffix r = raw, followed by the replicate number. For instance: 00hr_r1 = zero hours, raw data, replicate 1. Worksheet 2—“filter_normal”—contains the normalized read counts (akin to reads per kilobase per million [RPKM], adjusted for mapability) for all gene models (column header = “locus_tag”) in the *D. discoideum* genome. Column headings indicate the time point, followed by the suffix n = normal, followed by the replicate number 1 or 2. For instance: 00hr_n1 = zero hours, normalized data, replicate 1. Worksheet 3—“filter_average”—contains the normalized read counts for all gene models (column header = “locus_tag”) in the *D. discoideum* genome, averaged among replicates. Column headings indicate the time point, followed by the suffix avg = average. For instance: 00hr_avg = zero hours, averaged read count. Additional file 3:
**Contains three worksheets with data from the suspension experiment: Worksheet 1—“suspension_raw”—contains the raw read counts for all gene models (column header = “locus_tag”) in the** ***D. discoideum*** **genome.** Column headings indicate the time point, followed by the suffix r = raw, followed by the replicate number. For instance: 00hr_r1 = zero hours, raw data, replicate 1. Worksheet 2—“suspension_normal”—contains the normalized read counts (akin to reads per kilobase per million [RPKM], adjusted for mapability) for all gene models (column header = “locus_tag”) in the *D. discoideum* genome. Column headings indicate the time point, followed by the suffix n = normal, followed by the replicate number 1 or 2. For instance: 00hr_n1 = zero hours, normalized data, replicate 1. Worksheet 3—“suspension_average”—contains the normalized read counts for all gene models (column header = “locus_tag”) in the *D. discoideum* genome, averaged among replicates. Column headings indicate the time point, followed by the suffix avg = average. For instance: 00hr_avg = zero hours, averaged read count. Additional file 4:
**Contains a worksheet listing the 8,040 genes that met the minimum expression threshold in both filter and suspension experiments, and were thus included in PCA and MDS analyses.**
Additional file 5:
**(filter development) Contains the differential expression analysis false discovery rate (FDR) values determined by baySeq for all genes at all time point differences of k = 1-, 2-, 3-, and 4-hour, or “k-hops”.** We analyzed all-versus-all time points, but only used the 4-hour delta for all reported DE analyses. Due to constraints on the size of additional files, we have not included the baySeq output for the other time comparisons here. Further, due to the same size constraints, only the false discovery rate (FDR) for the “Differentially Expressed” model is given here. Other statistics, i.e. likelihood, and other models, i.e. “Not Differentially Expressed,” were also calculated. The complete baySeq output including all time point comparisons for both statistics and both models are available directly from the authors upon request. Additional file 6:
**(suspension) Contains the differential expression analysis false discovery rate (FDR) values determined by baySeq for all genes at all time point differences of k = 1-, 2-, 3-, and 4-hour, or “k-hops”.** We analyzed all-versus-all time points, but only used the 4-hour delta for all reported DE analyses. Due to constraints on the size of additional files, we have not included the baySeq output for the other time comparisons here. Further, due to the same size constraints, only the false discovery rate (FDR) for the “Differentially Expressed” model is given here. Other statistics, i.e. likelihood, and other models, i.e. “Not Differentially Expressed”, were also calculated. The complete baySeq output including all time point comparisons for both statistics and both models are available directly from the authors upon request. Additional file 7:
**Lists the differentially expressed genes (DDB_G identities) tabulated for each reference time point during the k-hop analysis.** This workbook contains four data worksheets. The name of each sheet describes the experiment “filter” or “suspension”, followed by “UP” or “DOWN”, indicating the direction of change in differential gene expression. In each sheet, the columns correspond to reference time points, and either the list of “gradual” or “rapid” DE genes, as tabulated in the k-hop analysis (Figure 2 in the main text). Additional file 8:
**Provides the gene ontology (GO) enrichment output for each of the DE gene lists in Additional file** **7** **, above.** This workbook contains four data worksheets. The name of each sheet describes the experiment “filter” or “suspension”, followed by “UP” or “DOWN”, indicating the direction of change in differential gene expression.

## Abbreviations

AU: Approximately unbiased (p-values)
cAMP: Cyclic adenosine monophosphate
DE: Differential expression
DIF: Differentiation inducing factor
FDR: False discovery rate
GABA: Gamma-aminobutyric acid
GO: Gene ontology
MDS: Multidimensional scaling
PC: Principal component
PCA: Principal component analysis
ROS: Reactive oxygen species
RPKM: Reads per kilobase per million
SDF: Spore differentiation factor
