# Supplementary material for: Leaps and lulls in the developmental transcriptome of Dictyostelium discoideum
Source: BMC Genomics. 2015 Apr 13;16(1):294. doi: 10.1186/s12864-015-1491-7 (PMC4403905; doi:10.1186/s12864-015-1491-7)
Supplement: Additional file 1: — Contains the supplementary results, Additional file 1 : Figures S1 – S10 and Additional file 1 : Tables S1 – S3 as referenced in the text. [file 12864_2015_1491_MOESM1_ESM.pdf]

Supplementary Results

Additional Observations from Principal Component Analysis

Despite their similar trajectories, the filter and suspension time courses displayed different spacing on the principal component (PC) 1 axis. For example, in the suspension experiment we observed a large separation of the last 3 time points from all the rest, coincident with a shift in the cAMP treatment from frequent pulses at low concentration to bulk high concentration. Such a large change was not observed in the filter development probably because the intrinsic transition from cAMP pulses to high constant cAMP levels is more gradual. We also noted a clear separation between the filter development and the suspension samples on the PC2 axis (Figure 1). Principal components are abstract in terms of biology, but they can sometimes be related to biological processes. PC1, for example, was roughly co-linear with time (data not shown), suggesting that it describes cumulative increasing temporal variation in the transcriptome. Such variation could be attributed to genes whose transcripts vary monotonically (either accumulating or diminishing) during development, or to cascades of temporally dependent changes in gene expression. One could speculate that separation between experiments on the PC2 axis reflects physical contact between cells, contact between cells and the substratum or any other substantive difference between treatments.

Since both experiments' transcriptional profiles followed similar principal component trajectories, we wanted to test their similarity more directly. We used linear regression modeling to test how well one experiment could predict the time series of the other. When the model was constructed using the filter data, it predicted the order of suspension time points with 93.8% accuracy (Spearman's correlation). On the other hand,

when trained on the suspension data, the model predicted the filter time sequence with 88.2% accuracy. The difference in model performance is likely due to the difference in time scales and sample number between experiments. Nevertheless, the high degree of correlation suggests that the filter data included a fairly complete subset of the transcription events of the suspension cells, while the suspension data represented a substantial but less complete picture of developmental gene expression. This finding is consistent with the fact that the suspension system can only mimic the first half of the developmental program.

Rapid shifts in transcriptional state are interspersed among intervals of gradual change

The transcriptomes we have analyzed reflect the population average of the behaviors of individual cells. Among the time points clustered closely together, mRNA abundance should change gradually. Therefore, when we observe large transcriptional shifts we can infer that most of the cells in the population must have experienced similar changes in gene expression. Gaps in the 2D trajectories (Figure 1, Supplementary Figure 2) might be viewed as intervals of synchronized changes among developing cells. We hypothesized that time intervals corresponding to the biggest gaps between clusters would contain the most differentially expressed (DE) genes. To test that hypothesis, we examined every two adjacent time points and found that only a fraction of these comparisons contained any DE genes at our significance threshold ($FDR \leq 0.01$) (Supplementary Figure 3). As predicted, these time points corresponded to the largest gaps in the transcriptome trajectories. For example, the largest number of up-regulated

genes in the filter development is at 12 hours (Supplementary Figure 3), which corresponds to the large gap between the 11h and 12h time points in the 2D plots (Figure 1, Supplementary Figure 2). The sparseness of DE genes at other time points was not an artifact of an overly stringent statistical threshold (Supplementary Figure 4), suggesting it is biologically significant. We therefore propose that much of development is accompanied by rather gradual changes in gene expression.

To identify which and how many transcripts changed in abundance at varying time scales, we took a "k-hop" approach to count the number of DE genes for all possible k = 1-, 2-, 3- and 4-hour comparisons (Additional Data Files 5, 6 and 7). This analysis confirmed that many genes were differentially expressed in the filter and suspension experiments at time deltas greater than 1 hour (Supplementary Figure 5A, 5C, Supplementary Table 2). In the filter data, we observed two major periods of down-regulation. The first one began at the onset development, when the cells just began to starve, and the second one began around 16h of development, when the multicellular structures were at the finger stage of development. We expected the down-regulation associated with starvation, but were surprised to count so many down-regulated genes between the 16h and 20h time points. Periods of up-regulation were more evenly distributed throughout development. In the suspension experiment, up- and down-regulation were rather evenly distributed. In both experiments, however, the majority of DE genes were detected at longer time distances, implying their expression changed gradually.

We compared the extent of rapid versus gradual up-regulation at each time point by re-plotting the k-hop results as the proportion of DE genes counted in the 1- and 2-

hour comparisons versus the 3- and 4-hour comparisons (Supplementary Figure 5B, 5D). This view illustrated time distances with rapid shifts in mRNA abundance (blue bars) interspersed between longer distances with more gradual change (red bars). The time points with the greatest proportion of rapid DE genes coincided with the largest shifts in the 2D trajectories. Specifically, at time points 12h and 18h in the filter series, over half of the DE genes had been up-regulated in the past two hours. Together these observations support our interpretation that the structure of the transcriptional trajectories reflects the underlying pace of change in gene expression. Transcript accumulation during early development was mostly gradual, while rapid differential expression was observed at the transition between aggregates and multicellular mounds, and in later development near the onset of culmination.

Supplementary Figure 1

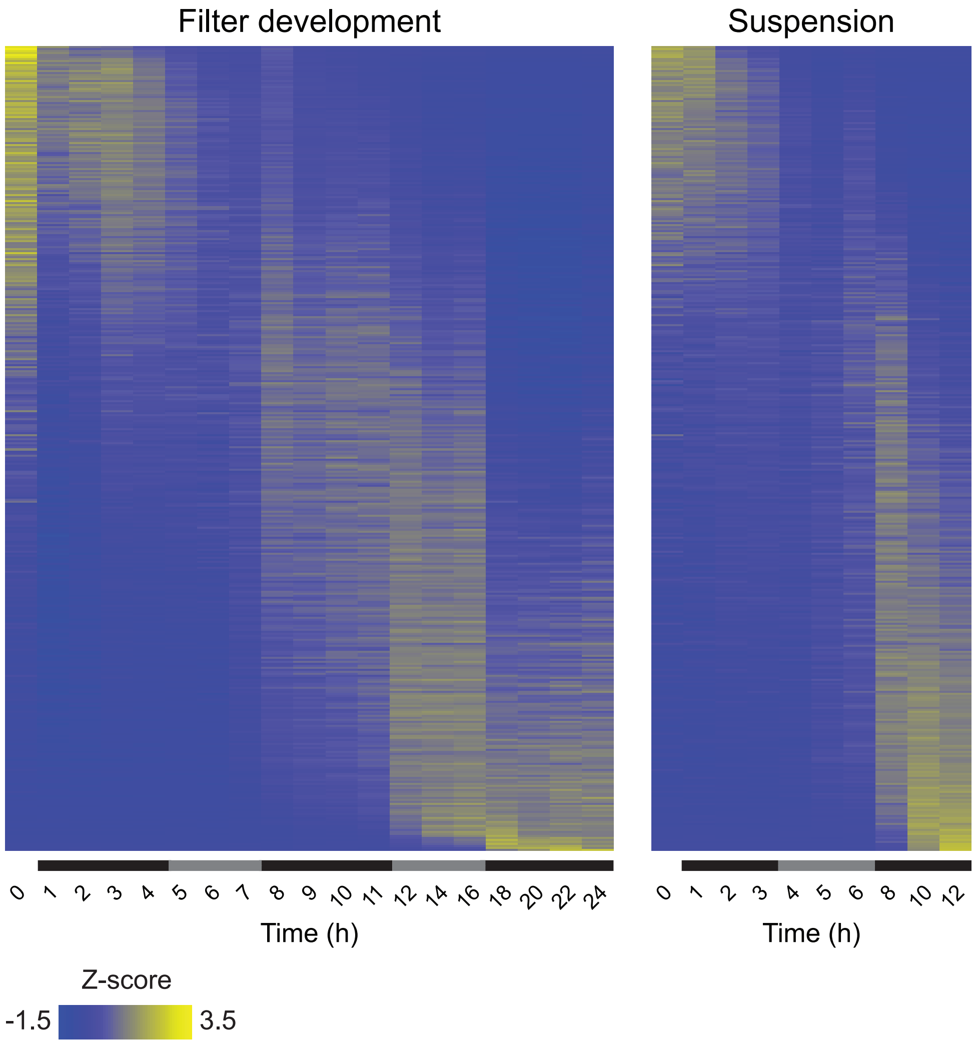

Supplementary Figure 2

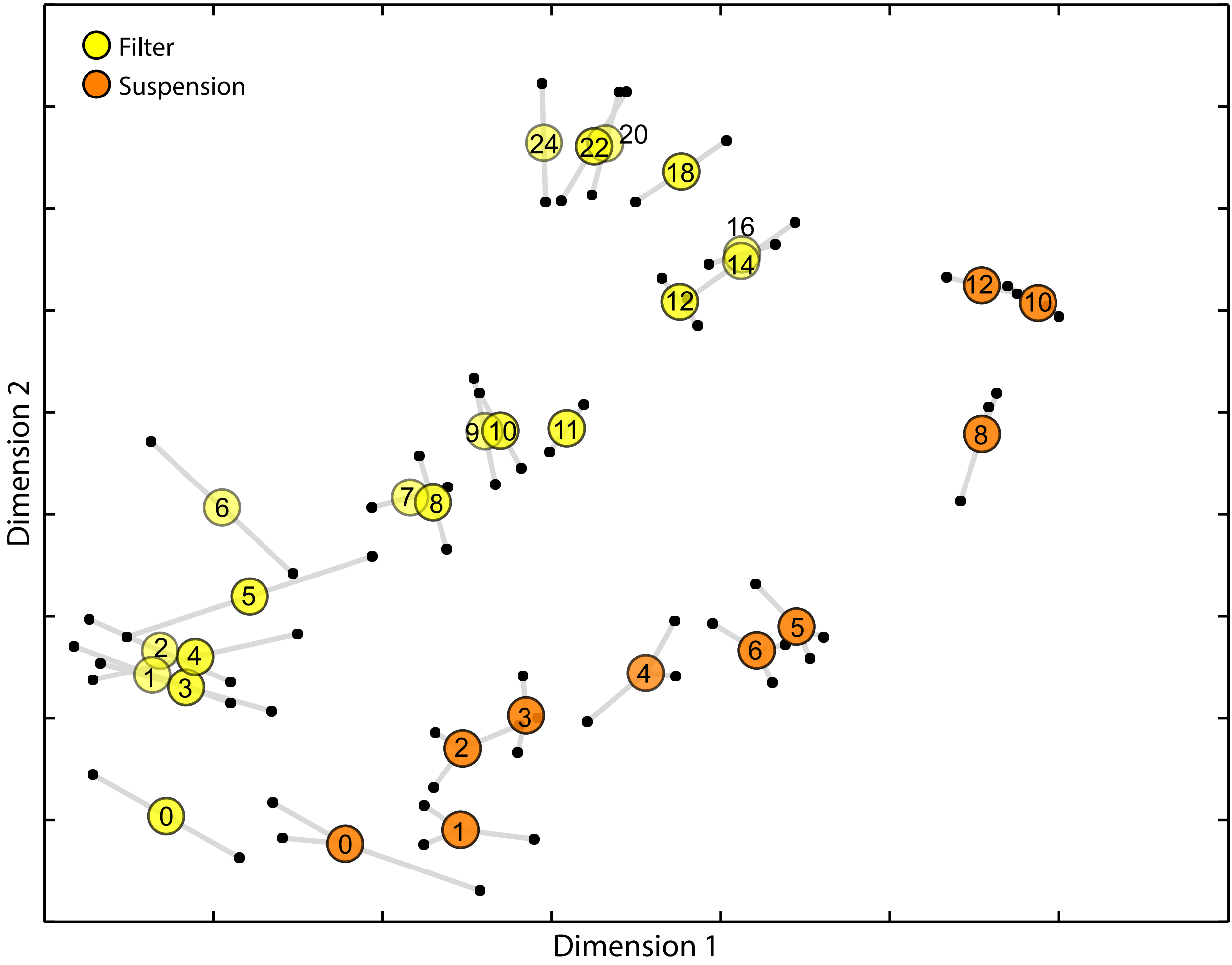

Supplementary Figure 3

A.

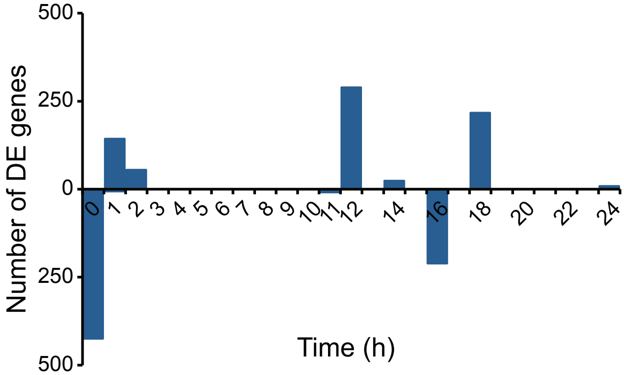

B.

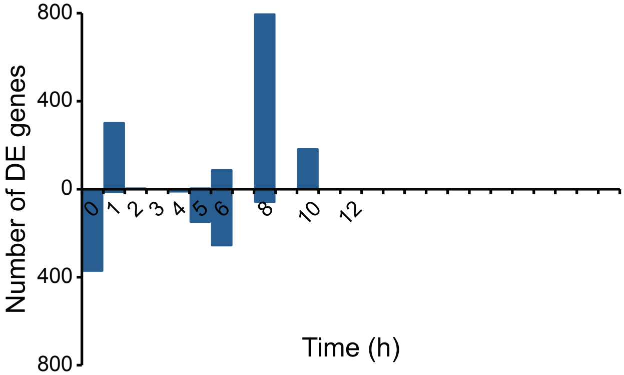

Supplementary Figure 4

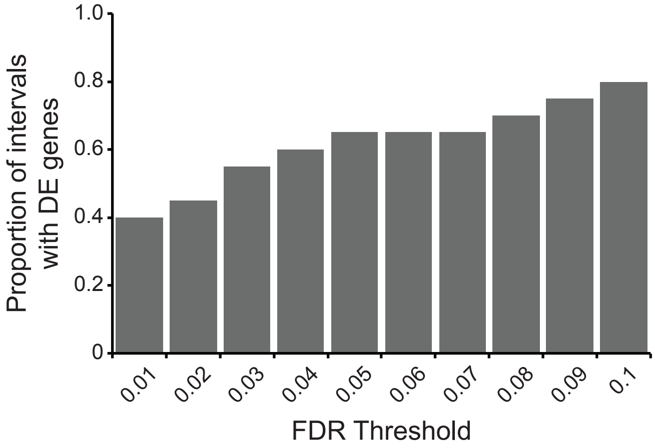

Supplementary Figure 5

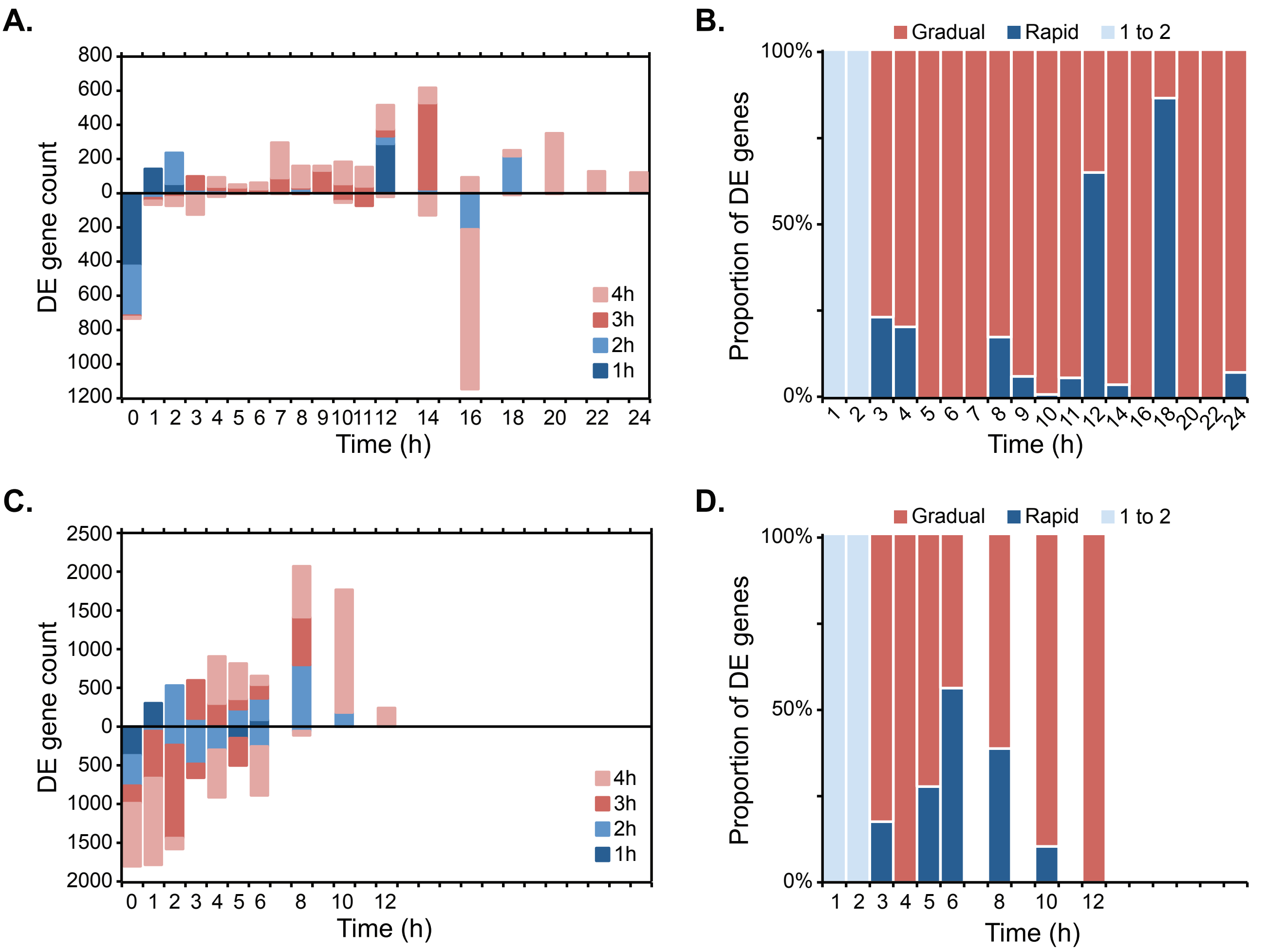

Supplementary Figure 6

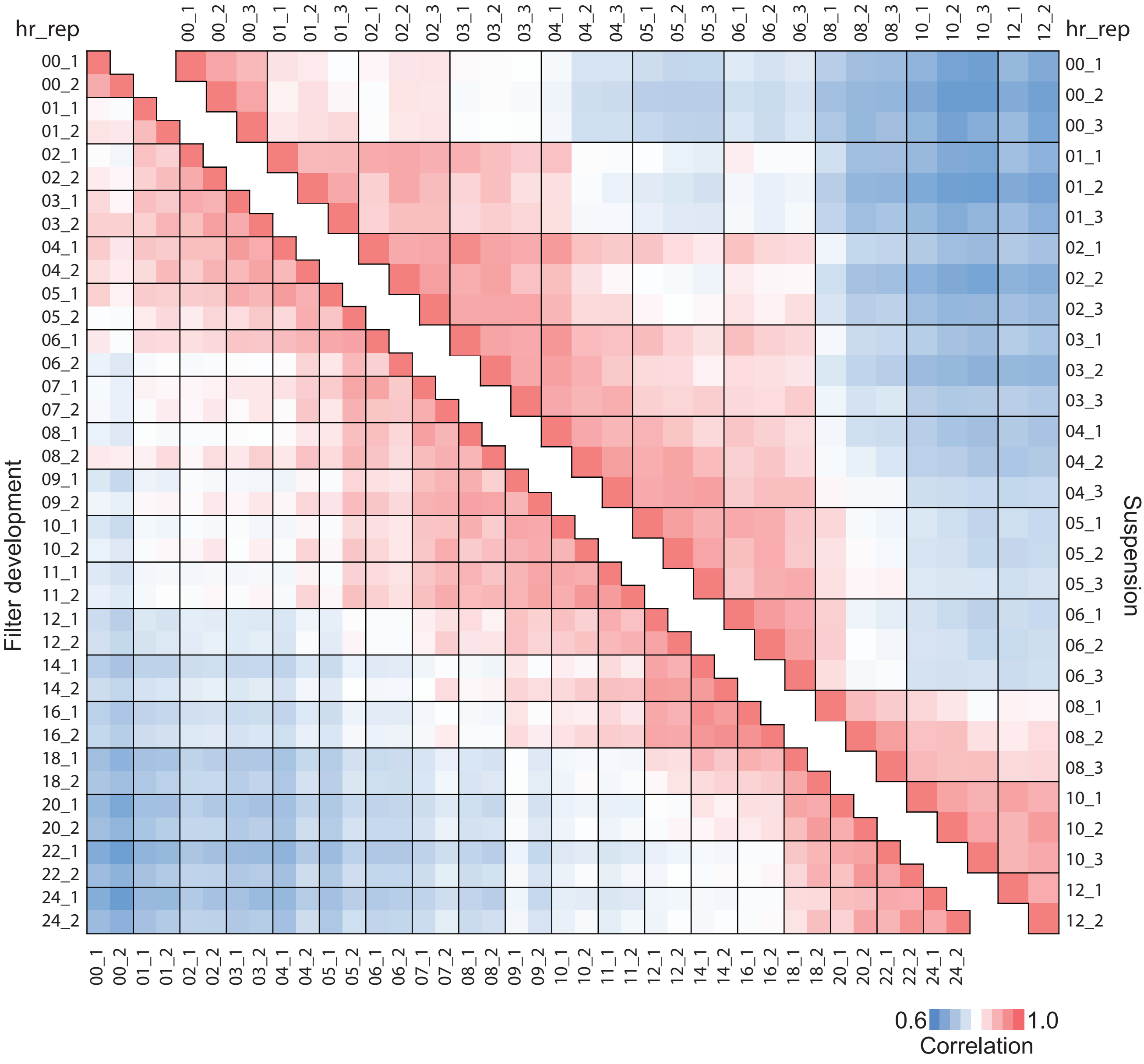

Supplementary Figure 7

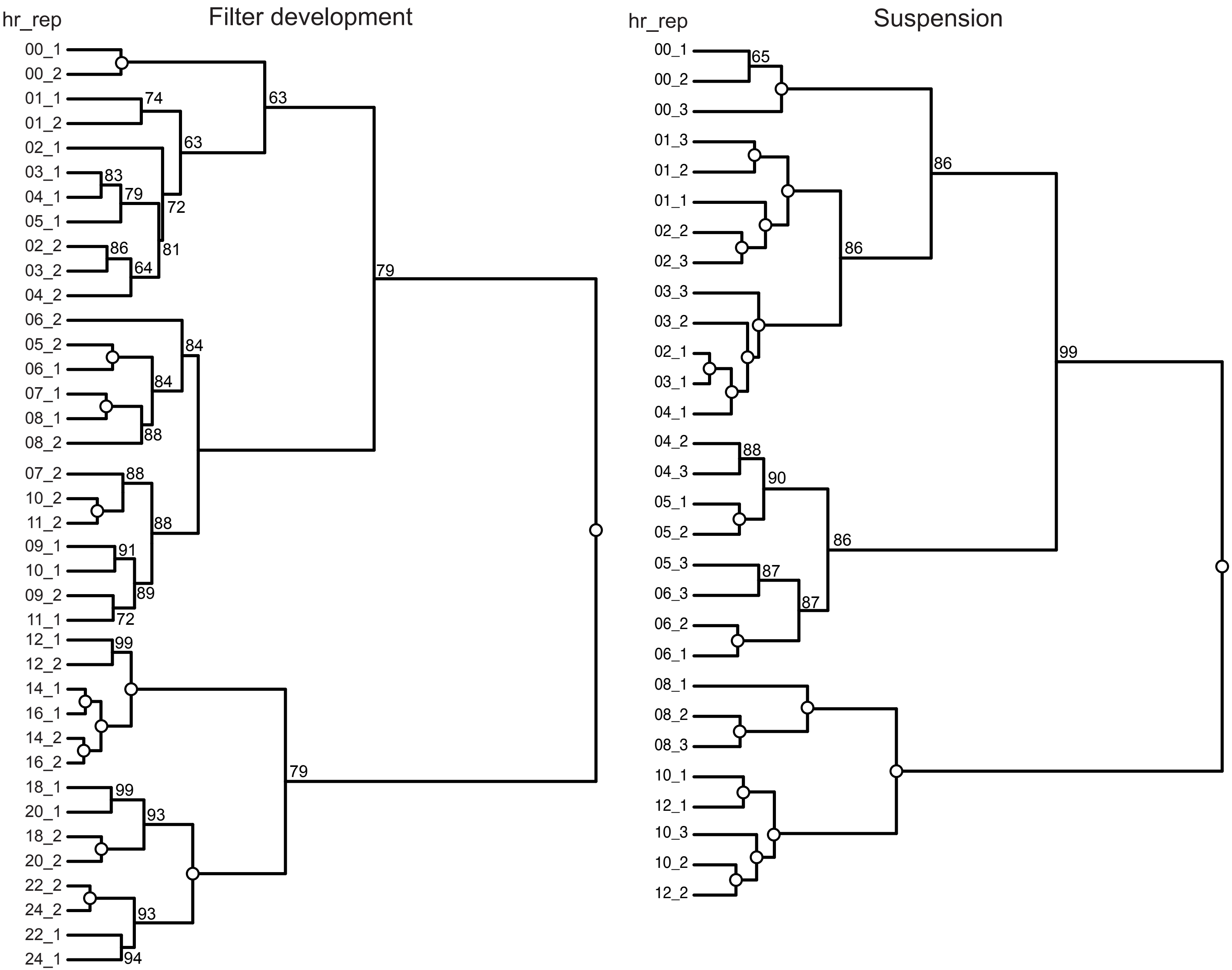

Supplementary Figure 8

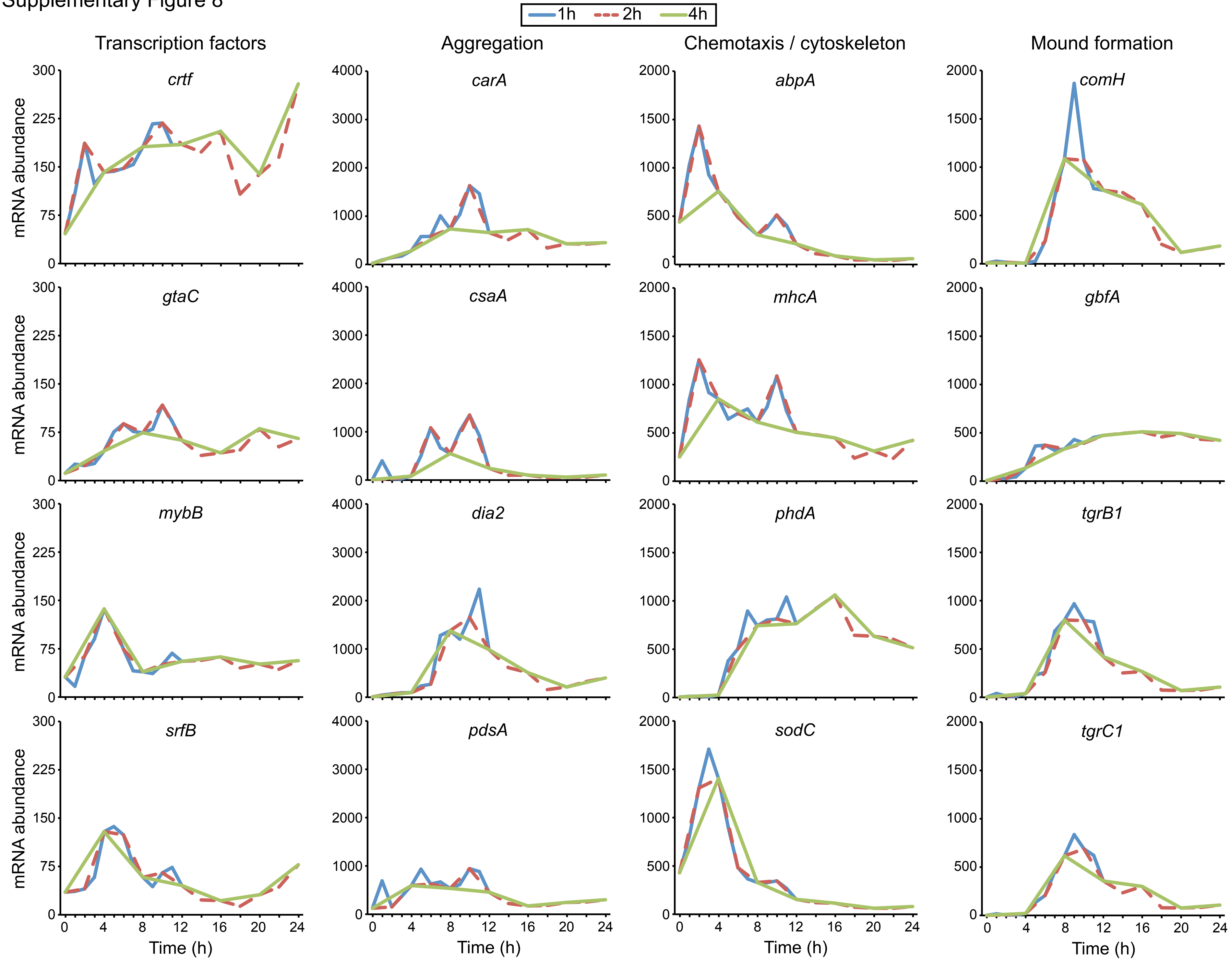

Supplementary Figure 9

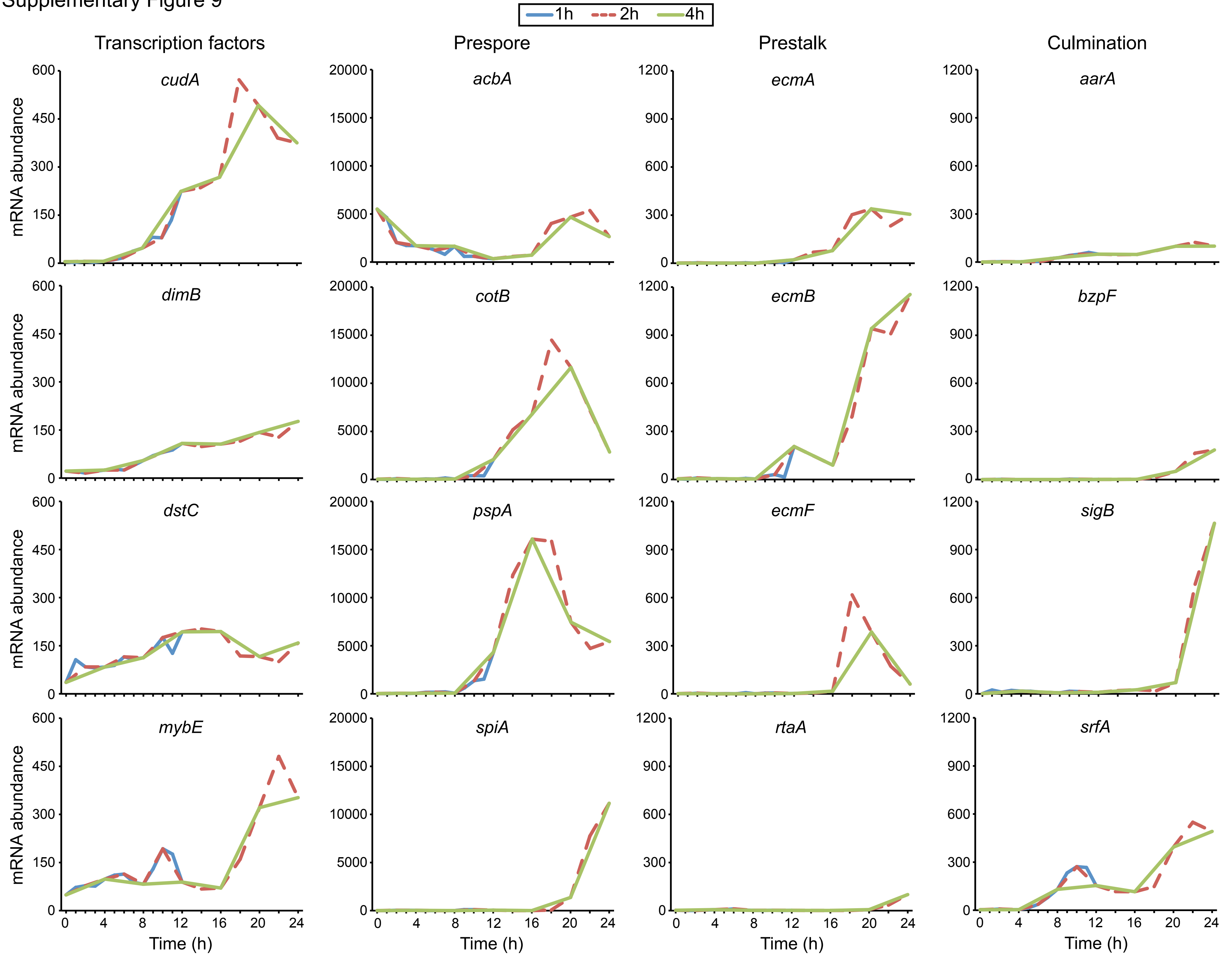

Supplementary Figure 10

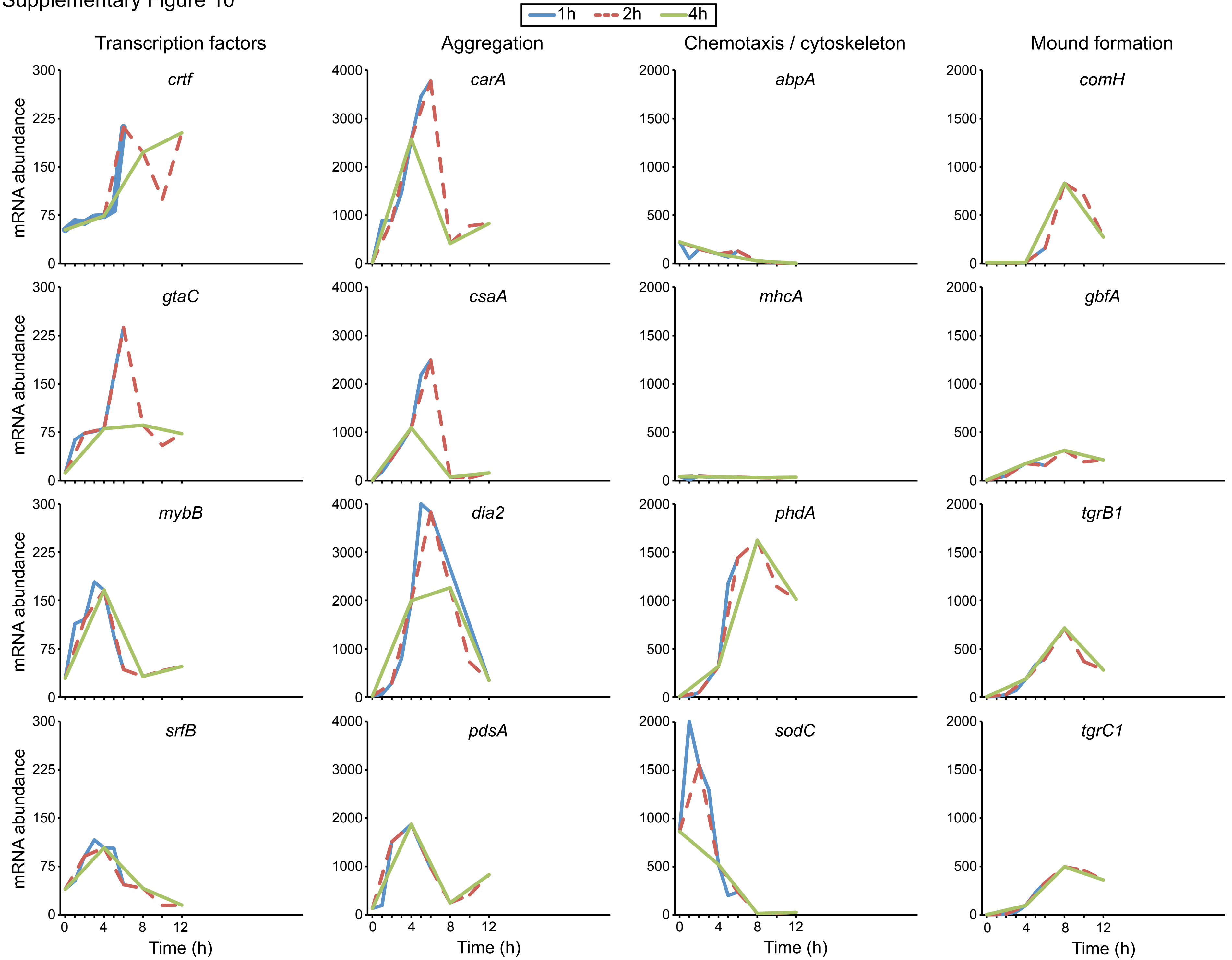

Supplementary Figure 1. Major shifts in developmental gene expression are apparent across the transcriptome. mRNA abundance is represented as Z-scores for all genes with at least 30 reads in one sample as indicated by the legend below the plot (8,040 genes intersecting both data sets). The filter development experiment is shown on the left and the liquid suspension experiment on the right. Each column represents a time point (hours), as indicated below the chart, and each row represents the average of 20 genes. The rows have been sorted by hierarchal clustering across both datasets, and as such, are in the same order in both heatmaps. Black and gray bars beneath the heatmaps indicate consecutive time point intervals that appear to be similar.

Supplementary Figure 2. Uneven developmental progression is revealed by multidimensional scaling of transcriptome time courses. We performed multi-dimensional scaling (MDS) on the transcriptome data from both filter development (yellow) and liquid suspension (orange) time courses, as with the PCA in Figure 1. The distance between any two points is correlated with the similarity between those data—the closer any two points, the more similar they are. The filter series contains two replicates of 19 time points and the suspension series contains two replicates with 10 time points and a third replicate with 9 time points (missing hour 12). For every time point we projected each sample transcriptome as a small black circle connected by whiskers to the other replicate(s). Large colored circles are placed at the center of the transcriptome projection replicates. The axes dimensions are arbitrary and unit-less.

Supplementary Figure 3. Few sequential time points contained differentially expressed genes. We scored differential expression (DE) for each gene compared between every two sequential time points. As in Figure 2, up-regulated DE genes were

counted looking back in time while down-regulated DE genes were counted looking forward in time. Time in hours is shown on the x-axis. On the y-axis, all values are positive—counts of up-regulated genes are displayed above the x-axis, while down-regulated counts extend below the x-axis. The data for filter development are shown in (A) and the data for liquid suspension are shown in (B). Note, the scale of the vertical axes varies between (A) and (B).

Supplementary Figure 4. Occurrence of differential expression changed little relative to false discovery rate. The proportion of sequential time point comparisons in which any genes were DE was tabulated (y-axis) for increasing FDR thresholds (x-axis). The slope of DE occurrence with respect to relaxing the FDR is shallow, with less than a 2-fold increase in DE gene presence over a 10-fold range in statistical significance.

Supplementary Figure 5. Rapid shifts are interspersed among gradual changes in mRNA abundance. We performed differential expression (DE) analysis for all genes between all time points in the filter development (A, B) and in the liquid suspension (C, D) experiments. The DE genes were counted for all 1-, 2-, 3- and 4-hour time comparisons (k-hops) (A, C). For up-regulation, DE genes were counted looking back in time—that is, we asked: of the genes with transcripts present at time = h, how many have accumulated significantly in the last k = 1, 2, 3 or 4 hours? Down-regulated genes were counted looking forward in time, asking: at time = h, how many genes will exhibit a reduction in transcript abundance in the next 1 to 4 hours? Genes were only counted once per reference time point for the smallest time delta in which they appeared. For example, if a gene was DE between 12h and 11h, as well as between 12h and 10h, it was placed in the 1-hour bin. Time in hours is shown on the x-axis and the number of genes is on the y-

axis. On the y-axis, all values are positive—counts of up-regulated genes are displayed above the x-axis and down-regulated genes are displayed below the x-axis. Transcript accumulation (up-regulation) was deemed "rapid" if genes were DE in either the 1- or 2-hour bin, or "gradual" if the genes were only found in the 3- or 4-hour comparison (B, D). The "1 to 2" category refers to the first two hours of the time series in which it is impossible to look back more than two hours. The x-axes in (B) and (D) are not continuous, rather all time points sampled in the filter series are adjacent. No samples were collected beyond 12 hours in the suspension treatment.

Supplementary Figure 6. High reproducibility between replicates and transcriptome similarities between nearby time points. We calculated the Spearman's rank correlations between the transcriptomes of all samples for each experiment and displayed these as an all-versus-all heatmap. Darker red boxes indicate higher correlations (more similarity), while darker blue boxes indicate lower correlations (less similarity), as indicated in the legend below the chart. Each sample is labeled according to its time point (hr) and replicate (rep): for example, '00_1' indicates the 0-hour time point of replicate 1. The data for filter development are shown on the left and the data for suspension are shown on the right, as indicated.

Supplementary Figure 7. Transcriptomes clustered with nearby time points within and between replications. We performed hierarchal clustering using Spearman's rank correlation for distance matrices (1 minus absolute value of correlation) to construct a dendrogram revealing the relationships between the transcriptomes. Each sample is labeled according to its time point and replicate as in Supplementary Figure 3. Approximately Unbiased (AU) p-values (%), computed by multiscale bootstrap

resampling, are given at each node, with open circles representing 100%. Higher AU values indicate greater confidence in a given branch point. The data for filter development are shown on the left and the data for suspension are shown on the right, as indicated.

Supplementary Figure 8. Transcription profiles of many early developmentally active genes depend on temporal resolution. The standardized mRNA abundance (y-axis) of the 16 early developmentally regulated genes shown in Figure 4 is plotted versus time (hours, x-axis). Data are from the filter development experiment. For each gene, expression values are included for time points at 1-, 2- and 4-hour intervals, as indicated in the legend above the plots. Each data point represents the average of 2 independent biological replicates. The y-axis scale is constant within, but varies between, functional categories (columns).

Supplementary Figure 9. Transcription profiles of some but not all late developmentally active genes depend on temporal resolution. The standardized mRNA abundance (y-axis) of the 16 late developmentally regulated genes shown in Figure 5 is plotted versus time (hours, x-axis). Data are from the filter development experiment. For each gene, expression values are included for time points at 1-, 2- and 4-hour intervals, as indicated in the legend above the plots. Each data point represents the average of 2 independent biological replicates. The y-axis scale is constant within, but varies between, functional categories (columns).

Supplementary Figure 10. For cells in suspension, changes in abundance of cAMP-responsive transcripts are revealed by more frequent sampling. The standardized

mRNA abundance (y-axis) of the 16 early developmentally regulated genes shown in Figure 4 is plotted versus time (hours, x-axis). Data are from the suspension treatment experiment. For each gene, expression values are included for time points at 1-, 2- and 4-hour intervals, as indicated in the legend above the plots. Each data point represents the average of 3 independent biological replicates. The y-axis scale is constant within, but varies between, functional categories (columns).

Supplementary Table 1. Sampling effort and sequencing output.

	Filter development	Suspension
Replicates	2	3
Samples per replicate	19, 19	10, 10, 9 [a]
Total samples	38	29
Reads per sample, median	7.8 Million (M)	14.8 M
Reads per sample, range	4.2 – 9.8 M	9.2 – 20.4 M
Gene models with mapped reads	12869	12869

[a] Missing 12-hour time point of replicate 3.

Supplementary Table 2. Numbers of differentially expressed genes identified in each experiment.

Direction of regulation	Counted	Filter	Suspension
Up	Single time point	912	2038
	Multiple occurrences	1154	2120
	Total genes [a]	2066	4158
Down	Single time point	1842	1908
	Multiple occurrences	286	2216
	Total genes	2128	4124
Both [b]	Total genes	637	1942

[a] The total number of differentially expressed genes includes those counted once, at a single time point during development, and those DE at multiple time point comparisons.

[b] Genes that were significantly up-regulated at one time interval and down-regulated at a different time interval during the experiments.

Supplementary Table 3. Developmental genes profiled in Figures 2, 3 and 4.

Gene symbol	Locus tag	Developmental process[a,b]	Gene product description[b]
aarA	DDB_G0288877	Culmination	Aardvark, beta-catenin related protein
abpA	DDB_G0268632	Chemotaxis/ Cytoskeleton	Actin binding protein
acbA	DDB_G0270658	Prespore	Acyl-CoA binding protein
bzpF	DDB_G0279529	Culmination	Basic-leucine zipper (bZIP) TF
carA	DDB_G0273397	Aggregation	cAMP receptor 1
comH	DDB_G0280547	Mound formation	GATA zinc finger domain-containing protein 2
cotB	DDB_G0276761	Prespore	Spore coat protein SP70
crtf	DDB_G0278077	Early TF	CarA transcription factor
csaA	DDB_G0289073	Aggregation	Contact site A cell-adhesion molecule
cudA	DDB_G0284465	Late TF	Culmination defective trancriptional regulator
dia2	DDB_G0291253	Aggregation	Differentiation associated protein 2
dimB	DDB_G0291372	Late TF	DIF Insensitive mutant B, bZIP TF
dstC	DDB_G0293532	Late TF	Dictyostelium signal transducer and activator of transcription family (STAT) protein C
ecmA	DDB_G0277853	Prestalk	Extracellular matrix protein ST430
ecmB	DDB_G0269132	Prestalk	Extracellular matrix protein ST310
ecmF	DDB_G0291291	Prestalk	Extracellular matrix, cellulose-binding domain-containing protein
gbfA	DDB_G0288755	Mound formation	G-box binding factor
gtaC	DDB_G0277589	Early TF	GATA zinc finger domain-containing protein 3
mhcA	DDB_G0286355	Chemotaxis/ Cytoskeleton	Myosin II heavy chain
mybB	DDB_G0275445	Early TF	Myb domain-containing protein
mybE	DDB_G0281969	Late TF	Myb domain-containing protein
pdsA	DDB_G0285995	Aggregation	cAMP phosphodiesterase
phdA	DDB_G0285845	Chemotaxis/ Cytoskeleton	PH domain-containing protein
pspA	DDB_G0267412	Prespore	Prespore specific, ponticulin-related, surface glycoprotein PsA precursor
rtaA	DDB_G0271852	Prestalk	Resistance to 7-aminocholesterol, lipid-translocating exporter family protein
sigB	DDB_G0293364	Culmination	Peptidase M8, leishmanolysin family protein
sodC	DDB_G0282993	Chemotaxis/ Cytoskeleton	Superoxide dismutase
spiA	DDB_G0289075	Prespore	Spore coat protein
srfA	DDB_G0281387	Culmination	Serum response factor MADS-box TF
srfB	DDB_G0282835	Early TF	Serum response factor MADS-box TF
tgrB1	DDB_G0280689	Mound formation	Transmembrane, IPT, IG, E-set, Repeat
tgrC1	DDB_G0280531	Mound formation	Transmembrane, IPT, IG, E-set, Repeat

[a] Biological process as simplistically catergorized in Figures 3 and 4

[b] Transcription factor (TF)
